# Assessment of the mode of action underlying development of liver lesions in mice following oral exposure to HFPO-DA and relevance to humans

**DOI:** 10.1093/toxsci/kfad004

**Published:** 2023-01-11

**Authors:** Melissa M Heintz, Laurie C Haws, James E Klaunig, John M Cullen, Chad M Thompson

**Affiliations:** ToxStrategies, LLC, Asheville, North Carolina 28801, USA; ToxStrategies, LLC, Austin, Texas 78759, USA; School of Public Health, Indiana University, Bloomington, Indiana 47405, USA; North Carolina State University College of Veterinary Medicine, Raleigh, North Carolina 27606, USA; ToxStrategies, LLC, Katy, Texas 77494, USA

**Keywords:** mode of action (MOA), liver, peroxisome proliferator-activated receptor α, (PPARα), HFPO-DA (GenX), PFAS

## Abstract

HFPO-DA (ammonium, 2,3,3,3-tetrafluoro-2-(heptafluoropropoxy)propanoate) is a short-chain polyfluorinated alkyl substance (PFAS) used in the manufacture of some types of fluorinated polymers. Like many PFAS, toxicity studies with HFPO-DA indicate the liver is the primary target of toxicity in rodents following oral exposure. Due to the structural diversity of PFAS, the mode of action (MOA) can differ between PFAS for the same target tissue. There is significant evidence for involvement of peroxisome proliferator-activated receptor alpha (PPARα) activation based on molecular and histopathological responses in the liver following HFPO-DA exposure, but other MOAs have also been hypothesized based on limited evidence. The MOA underlying the liver effects in mice exposed to HFPO-DA was assessed in the context of the Key Events (KEs) outlined in the MOA framework for PPARα activator-induced rodent hepatocarcinogenesis. The first 3 KEs (ie, PPARα activation, alteration of cell growth pathways, and perturbation of cell growth/survival) are supported by several lines of evidence from both *in vitro* and *in vivo* data available for HFPO-DA. In contrast, alternate MOAs, including cytotoxicity, PPARγ and mitochondrial dysfunction are generally not supported by the scientific literature. HFPO-DA-mediated liver effects in mice are not expected in humans as only KE 1, PPARα activation, is shared across species. PPARα-mediated gene expression in humans produces only a subset (ie, lipid modulating effects) of the responses observed in rodents. As such, the adverse effects observed in rodent livers should not be used as the basis of toxicity values for HFPO-DA for purposes of human health risk assessment.

Per‐ and polyfluoroalkyl substances (PFAS) are a large anthropogenic family of organic fluorinated compounds that have been used for decades in a broad range of industrial and consumer applications or products. The multiplicity of uses for these compounds stems from their physical and chemical properties. These same properties that impart functionality in products/applications also make PFAS resistant to biodegradation, photo-oxidation, direct photolysis, and hydrolysis, resulting in persistence and detection in the environment (Lau *et al.*, 2007). Toxicity studies in rodents examining effects following oral exposure to these compounds indicate that the liver is the primary target of toxicity (Costello *et al.*, 2022). However, considering the structural diversity of PFAS (eg, carbon chain length, interchain linkages, and functional groups), the mode of action (MOA) may differ between PFAS. Recently, an expert review panel, convened to provide guidance on key questions in PFAS risk assessment, concluded that “all PFAS” should not be grouped together for the purposes of assessing human health risk, as environmental persistence alone is not an adequate reason for grouping. The expert panelists also generally agreed that assuming similar toxicity or equal potency across the diverse class of PFAS is inappropriate (Anderson *et al.*, 2022).

HFPO-DA (ammonium, 2,3,3,3-tetrafluoro-2-(heptafluoropropoxy)-propanoate; CASRN 62037-80-3) is a short-chain PFAS used as a polymerization aid in the manufacture of some types of fluorinated polymers. Although HFPO-DA is broadly considered the replacement for perfluorooctanoate acid (PFOA) (See Guo *et al.* (2021) for structural comparisons of HFPO-DA and other PFAS.), different fluoropolymer manufacturers have developed their own individual PFOA replacement polymerization aid chemistries (Wang *et al.*, 2013). HFPO-DA is not used or found in the myriad of applications that PFOA has been historically used in, such as firefighting foam, carpets, paper, textiles, and other industrial and consumer products. In contrast, the limited number of fluoropolymers manufactured using HFPO-DA are utilized in important technologies including semiconductor fluid handling, high purity chemical processing, aerospace and telecommunications cabling, renewable hydrogen production, and lithium-ion batteries in transportation (https://www.teflon.com/en/industries-and-solutions/industries; https://www.nafion.com/en/industries).

In comparison to longer chain counterparts (eg, PFOA) which are often bioaccumulative in tissues, HFPO-DA is rapidly eliminated and does not bioaccumulate in mammalian tissues (Gannon *et al.*, 2016). The rapid elimination of HFPO-DA is consistent across species, with similar clearance rates in monkeys, rats, and mice, except for an approximate 10-fold increase in clearance in female rats (Gannon *et al.*, 2016). Further, recent ecological bioaccumulation studies in plants and aquatic organisms also demonstrate that HFPO-DA is not bioaccumulative in these organisms either (Wang *et al.*, 2022b).

The toxicity database for HFPO-DA has been reviewed previously (Thompson *et al.*, 2019). Therein, it was shown that HFPO-DA is not genotoxic, but induced cancers in Sprague Dawley rats in a pattern consistent with the PPARα tumor triad of liver adenomas/carcinomas, testicular Leydig cell tumors and pancreatic acinar cell tumors (Corton *et al.*, 2018; Felter *et al.*, 2018; Klaunig *et al.*, 2003). Specifically, female rats developed liver tumors at 500 mg/kg-day HFPO-DA, whereas male rats, exposed up to 50 mg/kg-day, exhibited a small but statistically significant increase in pancreatic acinar cell adenomas/carcinomas and a slight elevation in Leydig cell tumors—both at 50 mg/kg-day (Caverly Rae *et al.*, 2015). Thus, the carcinogenicity profile for HFPO-DA is consistent with other PPARα activators, inducing changes in the 3 tissues in the rat associated with the tumor triad. Although the carcinogenicity of HFPO-DA has not been evaluated in mice, the histopathological responses to HFPO-DA observed in mouse livers from subchronic studies are consistent with other PPARα activators and have been recently reviewed by Thompson *et al*. (forthcoming).

The MOA for liver tumors in rodents induced by PPARα activators is well-established in the scientific literature (Corton *et al.*, 2014; Klaunig *et al.*, 2003), and has also been proposed as the MOA for liver tumors in rats exposed to PFOA (Klaunig *et al.*, 2012). The human relevance of each Key Event (KE) within the PPARα MOA was thoroughly evaluated by Corton *et al.* (2018), with authors concluding that only KE 1, PPARα activation, is shared across humans and rodents. Furthermore, within KE 1, only receptor activation and induction of lipid metabolism genes occur in both species and downstream cell proliferation signaling, a key and required event in the formation of hepatic tumors, occurs specifically in rodents (Corton *et al.*, 2018). Based on this evaluation, these authors concluded that tumors induced via the PPARα MOA are not relevant to humans (Corton *et al.*, 2018).

In a 2021 toxicity assessment of HFPO-DA, the United States Environmental Protection Agency (USEPA) developed chronic and subchronic reference doses (RfDs) based on liver effects in mice from a subchronic oral toxicity study. Despite the significant evidence for involvement of peroxisome proliferator-activated receptor alpha (PPARα) activation according to both molecular and histopathological responses in the liver following HFPO-DA exposure in both rats and mice, alternate MOAs have been hypothesized with little supporting evidence. These hypothesized MOAs include those that involve cytotoxicity, participation of other PPAR subtypes (eg, PPARγ), or mitochondrial dysfunction (USEPA, 2021). Importantly, the liver effects used to develop USEPA’s RfDs for HFPO-DA are part of the early KEs in the PPARα MOA. Herein, we reviewed toxicity data for HFPO-DA in mice from the primary peer-reviewed literature and USEPA’s Health and Environmental Research Online (HERO) database and assessed these data in the context of the first 3 KEs outlined in the PPARα MOA framework as described in Corton *et al.* (2014). Since the time of USEPA’s assessment, additional data important for informing the MOA for HFPO-DA has been generated, including caspase-3 immunohistochemical and transcriptomic analyses of mouse liver from the reproductive/developmental study used to develop USEPA’s RfDs for HFPO-DA (Heintz *et al.*, 2022; Thompson *et al*., forthcoming). This new information, along with existing information, including published hepatic transcriptomic results in mice for HFPO-DA from a 90-day subchronic study (Chappell *et al.*, 2020) were analyzed together and integrated herein to evaluate transcriptomic support for the early KEs of the PPARα MOA and alternate MOAs. The concordance of timing and dose-response of observed liver effects for HFPO-DA, biological plausibility, and human relevance are evaluated within the PPARα MOA framework.

## MOA analysis

**Figure 1.**
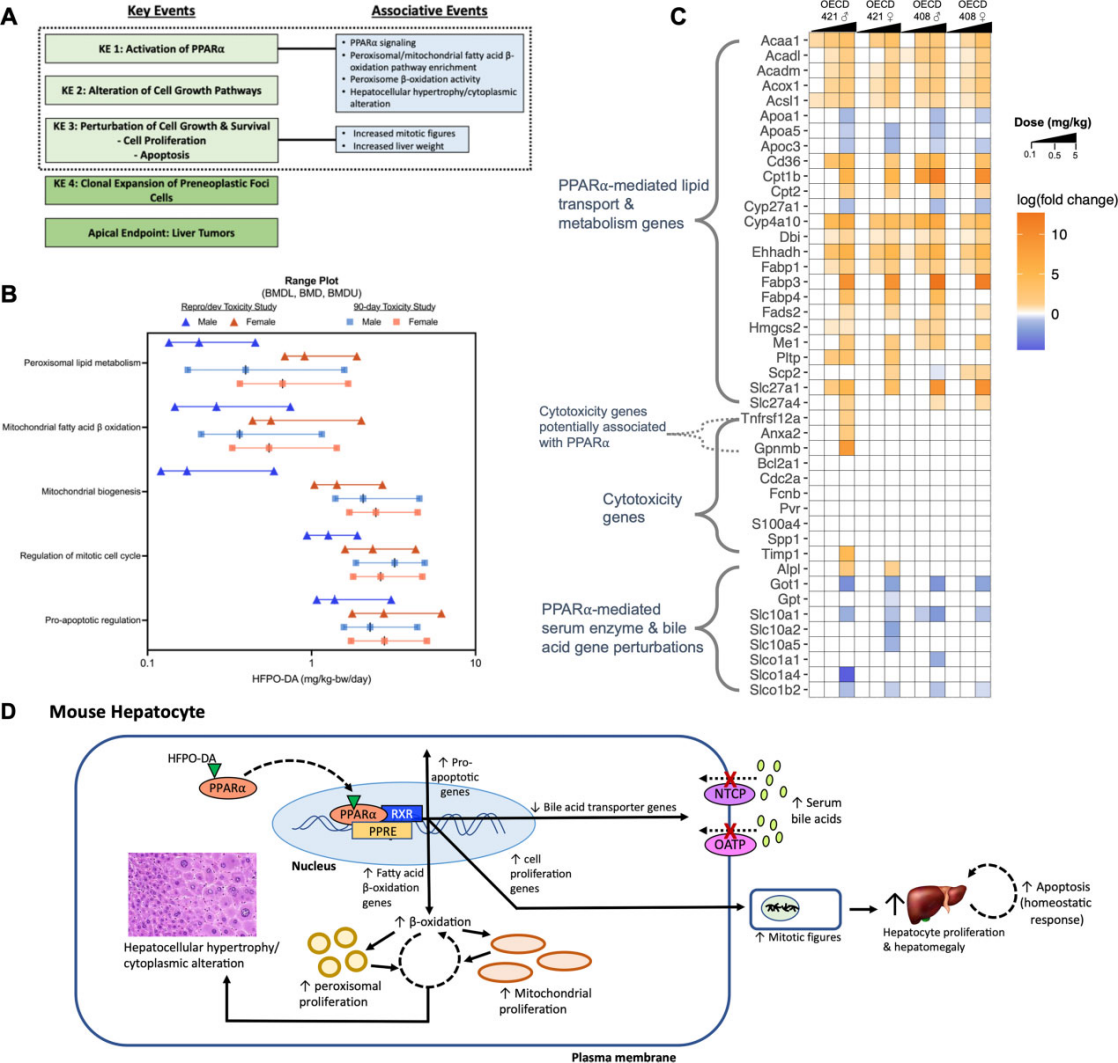
Transcriptomic concordance and support for the mode of action (MOA) underlying development of liver lesions in mice following oral exposure to HFPO-DA. A, Key and associative events of the PPARα MOA in rodents. Data supporting the key and associative events within the dashed box are evaluated herein for HFPO-DA. Key Events are empirical and observable causal precursor steps to the formation of liver tumors. Each sequential event is necessary, but not sufficient by itself in the absence of other Key Events, for the formation of liver tumors. Associative Events are biological endpoints that can be used as indicators or biomarkers of Key Events in the PPARα MOA. For the purposes of this current evaluation Key and Associative Events are not discerned. Adapted from Corton *et al.* (2014). B, Range plots (median BMDL, median BMD, and median BMDU) for select pathways containing dose-responsive genes in livers of HFPO-DA exposed male and female mice from Heintz *et al.* (2022) and Chappell *et al.* (2020). See Heintz *et al.* (2022) for additional benchmark dose modeling details. C, Heatmap of hepatic gene expression of selected genes included in PPARα-mediated lipid transport and metabolism (KEGG PPAR Signaling Pathway) or cytotoxicity (Corton* et al.*, 2020; Glaab *et al.*, 2021) gene sets, as well as genes encoding liver enzymes (*Alpl* (ALP), *Got1* (AST), and *Gpt* (ALT)) and bile acid transporters. Significant (FDR < 0.1) differentially expressed genes (rows) are indicated by green (up-regulated genes) or red (down-regulated genes) colors. Intensity of colors is based on the log(fold change) value of each gene for each study, sex, and dose group. White cells indicate gene was not significantly altered by HFPO-DA exposure compared to respective controls. Transcriptomic data included in this heatmap are from the OECD 421 reproductive/developmental toxicity and the OECD 408 90-day toxicity study for male and female mice. HFPO-DA dose levels are indicated by right triangles at the top of the heatmap for each study, increasing from 0.1 to 5 mg/kg for each study and sex. Methods for transcriptomic analyses are described in Chappell *et al.* (2020) and Heintz *et al.* (2022). D, Summary of the role of PPARα in the observed liver changes in mice exposed to HFPO-DA. Upon PPARα activation, solid arrows represent either subsequential PPARα-mediated or downstream events, and dashed arrows indicate positive feedback mechanisms.

To evaluate the evidence concerning the PPARα MOA for HFPO-DA in mice, we have characterized the evidence base for the first 3 KEs of the PPARα MOA framework described in Corton *et al.* (2014) (Figure 1A;Table 1).

**Table 1.** Supporting evidence for HFPO-DA within key events 1–3 of the MOA for PPARα activator-induced rodent liver tumors

Key Event 1: PPARα Activation				Key Event 2: Alteration of Cell Growth Pathways				Key Event 3: Perturbation of Cell Growth and Survival			
Effects	**Result** ^a^	Test System	References	Effect	**Result** ^a^	Test System	References	Effect	**Result** ^a^	Test System	References
PPARα receptor activation	**+**	Mouse PPARα reporter assay	Chappell *et al.* (2020)	Mitotic cell cycle signaling	**+**	Mouse oral 90-day	Chappell *et al.* (2020)	Increased liver weight	**+**	Mouse oral 90-day	DuPont (2010a)
		Rat PPARα reporter assay	Chappell *et al.* (2020)			Mouse oral repro/dev	Heintz *et al.* (2022)			Mouse oral repro/dev	DuPont (2010b)
			Evans *et al.* (2022)			Mouse oral 28-day	Guo *et al.* (2022)			Mouse oral GD 1.5–17.5	Blake *et al.* (2020)
PPARα signaling and peroxisomal/mitochondrial fatty acid β-oxidation gene expression/pathway enrichment	**+**	Mouse oral 90-day	Chappell *et al.* (2020)	Pro-apoptotic regulation	**+**	Mouse oral 90-day	Chappell *et al.* (2020)			Mouse oral 28-day	Haas (2008)
		Mouse oral repro/dev	Heintz *et al.* (2022)			Mouse oral repro/dev	Heintz *et al.* (2022)				Guo *et al.* (2021, 2022)
		Mouse oral 28-day	Guo *et al.* (2021, 2022)							Rat oral cancer bioassay	Caverly Rae *et al.* (2015)
			Wang *et al.* (2017)					Increased serum liver enzymes^b^	**−**	Mouse oral GD 1.5–17.5	Blake *et al.* (2020)
Peroxisome β-oxidation activity	**+**	Mouse oral 28-day	Thompson *et al.* (2019)						**+**	Mouse oral 28-day	Haas (2008)
											Guo *et al.* (2021)
		Rat oral 28-day	Thompson *et al.* (2019)							Mouse oral 90-day	DuPont (2010a)
Peroxisome proliferation	**+**	Mouse oral 90-day	Chappell *et al.* (2020)							Rat oral cancer bioassay	Caverly Rae *et al.* (2015)
		Mouse oral repro/dev	Heintz *et al.* (2022)					Increased mitotic figures	**+**	Mouse 28-day	Haas (2008)
		Mouse oral GD 1.5–17.5	Blake *et al.* (2020)							Mouse oral GD 1.5–17.5	Blake *et al.* (2020)
		Mouse oral repro/dev	Thompson *et al*. (forthcoming)							Mouse oral 90-day	NTP (2019), Chappell *et al.* (2020)
Hepatocellular hypertrophy/cytoplasmic alteration	**+**	Mouse 28-day	Haas (2008)							Mouse oral repro/dev	NTP (2019), Thompson *et al*. (forthcoming)
			Guo *et al.* (2021)					Increased caspase-3 (Apoptosis)	**+**	Mouse oral GD 1.5–17.5	Blake *et al.* (2020)
		Mouse oral GD 1.5–17.5	Blake *et al.* (2020)							Mouse oral 90-day	NTP (2019), Chappell *et al.* (2020)
		Mouse oral 90-day	DuPont (2010a), NTP (2019)							Mouse oral repro/dev	NTP (2019), Thompson *et al*. (forthcoming)
		Mouse oral repro/dev	DuPont (2010b), NTP (2019)								
		Rat oral cancer bioassay	Caverly Rae *et al.* (2015)								

GD, gestation day.

a + indicates positive findings and − indicates negative findings in HFPO-DA-exposed mouse or rat models.

b Putative marker of hepatocellular proliferation and PPARα-mediated bile acid perturbation in rodents.

### KE 1: PPARα activation

Broadly, evidence streams for PPARα activation (KE 1) include PPARα receptor binding and/or activation, increased expression of genes/proteins involved in fatty acid β-oxidation, increased palmitoyl-CoA oxidase activity, and morphological evidence of peroxisome proliferation (Corton *et al.*, 2018). In addition, analysis of mRNA or transcriptomic responses to PPARα activation, or the loss of any of the aforementioned effects in knockout studies, also provides evidence of PPARα activation. To date, we are not aware of any published studies on HFPO-DA in genetically modified rodent models.

HFPO-DA-mediated PPARα activation is supported by several lines of evidence (Table 1), including the activation of both mouse and rat PPARα receptors by HFPO-DA in *in vitro* reporter assays (Chappell *et al.*, 2020; Evans *et al.*, 2022). Activation of PPARα by HFPO-DA was measured by luciferase activity in reporter gene constructs, with a lower EC_50_, equal to 40 µM, observed for mouse PPARα, compared to an EC_50_ equal to 114 µM for rat PPARα (Chappell *et al.*, 2020). Investigations on the potency of PFAS activating rat PPARα found that HFPO-DA and HFPO-DA ammonium salt were the most potent among 16 PFAS studied, with EC_20_ values equal to 83.18 µM and 75.86 µM, respectively. Rat PPARα activation levels for all other PFAS tested (including PFOA) were too low compared to the positive control to calculate EC_20_ values. HFPO-DA was also a more potent activator of rat PPARα than the endogenous fatty acids, oleic acid (EC_20_ = 794.33 µM) and clofibric acid (EC_20_ = 562.34 µM) (Evans *et al.*, 2022). Triglycerides of oleic acid are the primary component of some vegetable oils, most notably olive oil (Boskou *et al.*, 2006).

Exposure of HFPO-DA for 28 days also increased hepatic peroxisome β-oxidation activity in both mice and rats (Thompson *et al.*, 2019). Although a significant increase in peroxisome β-oxidation activity was observed at HFPO-DA dose levels ≥ 3 mg/kg in mice dosed with 0, 0.1, 3, or 30 mg/kg for 28 days (Thompson *et al.*, 2019), no enzyme activity data are available at doses between 0.1 and 3 mg/kg. However, analysis of hepatic transcriptomic data in mice from a 90-day subchronic study by Chappell *et al.* (2020) and from a reproductive/developmental study by Heintz *et al.* (2022) demonstrate significant enrichment of the specific gene sets: KEGG peroxisome, REACTOME peroxisomal lipid metabolism, and REACTOME β-oxidation of very long chain fatty acids, at both 0.5 and 5 mg/kg HFPO-DA (Supplementary Table 1), with median benchmark doses (BMDs) for the latter REACTOME gene sets ranging between 0.39–0.67 and 0.24–0.34 mg/kg HFPO-DA, respectively (Heintz *et al.*, 2022). These results indicate induction of peroxisome β-oxidation by HFPO-DA at dose levels lower than 0.5 mg/kg in male and female mice.

Additional evidence for PPARα activation in the livers of HFPO-DA-exposed mice is also provided by the transcriptomic findings from Chappell *et al.* (2020) and Heintz *et al.* (2022). Hepatic transcriptomic data from these studies show significant enrichment of gene sets related to fatty acid metabolism, peroxisome proliferation, and PPAR signaling (ie, “KEGG PPAR Signaling Pathway” and “WP PPAR Signaling”). Although general PPAR signaling gene sets were among the most significantly upregulated in both studies, the specificity for PPARα activation by HFPO-DA was demonstrated by the differentially expressed genes that are specific to the α subtype and a general lack of changes in expression levels of most of the genes specific to other PPAR subtypes (Supplementary Figure 1). Importantly, although some publicly curated PPAR signaling gene sets (eg, KEGG) denote that gluconeogenesis genes are specifically regulated by PPARγ, which is likely the case for adipose tissue, several studies have determined PPARα as a key regulator of glucose metabolism in the liver (Kersten, 2014; Peeters and Baes, 2010). Thus, differential gene expression of *Pck1* and *Aqp7* in high dose groups across mouse studies in Supplementary Figure 1 likely indicates PPARα-mediated regulation of gluconeogenesis in the liver. Gene sets specific to the PPARα subtype were also significantly enriched in both male and female mice at 0.5 and 5 mg/kg HFPO-DA (Supplementary Table 1). In addition to the upregulation of genes that affect fatty acid uptake, activation and oxidation, HFPO-DA upregulated several *Pex* genes, involved in peroxisome biogenesis, in male and female mice at 0.5 and 5 mg/kg. Similarly, other research groups (Guo *et al.*,2021, 2022; Wang *et al.*, 2017) also determined that KEGG PPAR signaling and lipid metabolism gene sets were significantly enriched in male mice treated for 28 days with 0.4–10 mg/kg or 1 mg/kg HFPO-DA, respectively.

A recent review of rodent liver histopathology in response to PFAS noted that “PFAS-induced liver injury and steatosis may not depend on PPARα alone” (Costello *et al.*, 2022). However, a separate recent review of multiple HFPO-DA studies in mice found that most studies did not report steatosis and those that did were not convincing based on the published images (Thompson *et al*., forthcoming). Morphological evidence of peroxisome proliferation (eg, hepatocellular hypertrophy/cytoplasmic alteration) from HFPO-DA exposure was observed in H&E stained livers of mice from subchronic and reproductive/developmental toxicity studies (DuPont, 2010a,b; NTP, 2019). In addition, Blake *et al.* (2020) also reported an increased number of peroxisomes with increasing HFPO-DA dose in mice using transmission electron microscopy.

### KE 2: alteration of cell growth pathways

Evidence for altered cell growth pathways (KE 2) that has been observed in rodent liver following exposure to PPARα activators (Corton *et al.*, 2018; Morimura *et al.*, 2006) includes increased expression of genes that promote cell proliferation, such as c-myc, cyclin D1 (Cd1), cyclin-dependent kinase 1 (Cdk1), and cyclin-dependent kinase 4 (Cdk4). In addition, KE 2 may also include involvement of activation of nonparenchymal cells (eg, Kupffer cells) that, once activated, secrete cytokines such as tumor necrosis factor α (TNFα), interleukin-1α (IL-1α), and interleukin-1β (IL-1β) (Corton *et al.*, 2018).

Evidence for KE 2 following HFPO-DA exposure is supported by benchmark dose modeling (BMD) results of gene expression data from Chappell *et al.* (2020) and Heintz *et al.* (2022) in addition to liver histopathology data (Table 1) (NTP 2019). Functional classification of significant dose-responsive genes showed lower median BMDs are associated with fatty acid metabolism-related gene sets, whereas enriched gene sets with higher median BMDs are related to mitotic cell cycle (Figure 1B). These transcriptomic results are phenotypically anchored by H&E staining of liver sections, as mitotic figures were increased at the highest dose levels in both studies (Chappell *et al.*, 2020; NTP, 2019).

### KE 3: perturbation of cell growth and survival

Evidence for perturbation of cell growth and survival (KE 3) includes hepatocyte proliferation (increased cell number) and altered apoptotic rates, resulting in hepatocyte hypertrophy and subsequent liver enlargement. At high doses, potent PPARα activators exhibit sustained or chronic increases in cell proliferation (Peters *et al.*, 1998; Ward *et al.*, 1988).

Support for KE 3 is well established based on transcriptomic, histopathology, and liver weight data (Table 1). Relative liver weight significantly increased in male and female mice from subchronic studies for HFPO-DA (Blake *et al.*, 2020; DuPont, 2010a,b; Guo *et al.*, 2021, 2022). Mitotic figures have been observed in H&E stained mouse liver sections at 5 mg/kg HFPO-DA (Chappell *et al.*, 2020; DuPont, 2010a,b; NTP, 2019), as well as in the livers of mouse dams exposed to 2 and 10 mg/kg (Blake *et al.*, 2020). In addition, increased incidence of “single cell necrosis” has been reported in mice from 90-day and reproductive/developmental toxicity studies (DuPont, 2010a,b; NTP, 2019); however, a recent reevaluation of liver sections from these studies demonstrated that some hepatocytes putatively considered to be necrotic also stained with the apoptotic marker, activated caspase-3 (Thompson *et al*., forthcoming). As such, apoptotic cell death, as opposed to necrotic cell death, may be the predominant form of cell death in the livers of mice exposed to HFPO-DA.

Pro-apoptotic gene sets were also significantly enriched at higher dose levels in livers from HFPO-DA-exposed mice (Figure 1B) (Chappell *et al.*, 2020; Heintz *et al.*, 2022). This induction of pro-apoptotic gene expression is anchored to phenotypic evidence for hepatocellular apoptosis via H&E staining and caspase-3 immunostaining (Chappell *et al.*, 2020; Thompson *et al*., forthcoming). While PPARα activators are reported to suppress apoptosis under acute exposure scenarios, PPARα activators have also been reported to increase apoptosis in the livers of mice undergoing cell proliferation in repeat dose studies (Corton *et al.*, 2018). In addition, exposure to the PPARα activator, WY-14643, has been shown to induce some pro-apoptotic gene expression and repress some anti-apoptotic genes in wild type but not PPARα-null mice (Xiao *et al.*, 2006). These data suggest that PPARα might promote apoptotic signaling directly or as a homeostatic response to PPARα-related growth signals.

## Temporal and dose-response concordance of HFPO-DA liver effects with PPARα MOA

The U.S. EPA Guidelines for Carcinogen Risk Assessment and the IPCS advocated the adoption of the Bradford Hill criteria for assessing causality in epidemiological studies for application in judging the strength of data in supporting MOA analyses (Hill 1965; Sonich-Mullin *et al.*, 2001; USEPA 2005). More recently, inconsistencies in the use of these frameworks have been identified and clarified by development of so-called evolved Bradford Hill criteria by asking specific questions related to each criterion (Meek *et al.*, 2014). In addition to temporal concordance, each of these modified Hill criteria (ie, biological concordance, essentiality, concordance of empirical observations among KEs, consistency, and analogy) are evaluated below as they relate to the MOA for HFPO-DA-induced liver changes in mice.

The first evolved Bradford Hill consideration, biological concordance, determines whether the hypothesized MOA conflicts with broader scientific knowledge and additionally whether a MOA is well established (Meek *et al.*, 2014). In the case of the PPARα MOA for HFPO-DA presented herein, the KEs are consistent with established PPARα activators including other PFAS. As for how well the MOA is established, the PPARα MOA is well established for rodent liver tumors (Corton *et al.*, 2014; Felter *et al.*, 2018; Klaunig *et al.*, 2003). As previously noted, the analyses herein focus on the first 3 KEs because the non-neoplastic liver changes used to develop USEPA’s RfDs for HFPO-DA are a part of the early KEs in the PPARα MOA. Although chronic bioassays with HFPO-DA are not currently available in mice, chronic exposures in mice are expected to yield similar results as to what has been observed in rats (ie, liver tumors) (Caverly Rae *et al.*, 2015). As described in the MOA analysis above, HFPO-DA has been shown to cause sustained PPARα activation, altered cell growth and increased incidence of non-neoplastic lesions in the livers of mice. The PPARα MOA for HFPO-DA is supported by data consistent with the molecular biology of carcinogenesis (Hanahan and Weinberg, 2011) as well as data for other biological endpoints associated events not related to hepatocarcinogenesis, but related to PPARα-specific induction, such as peroxisome proliferation and the induction of lipid metabolism genes. Furthermore, increased hepatocyte and liver growth are fundamental features of tumor growth (Corton *et al.*, 2014). Therefore, the available data for HFPO-DA are consistent with the early KEs in the PPARα MOA for rodent hepatocarcinogenesis including hepatocellular hypertrophy (cytoplasmic alteration), apoptosis, and focal necrosis.

The second evolved Bradford Hill consideration, essentiality, examines whether KEs are reversible if dosing ceases or if a KE is prevented (Meek *et al.*, 2014). Although studies in PPARα-null mice are not yet available for HFPO-DA, the KEs are prevented/mitigated in studies conducted with classic PPARα activators or other PFAS in PPARα-null mice.

The third evolved Bradford Hill consideration, concordance of empirical observations, assesses the dose-response and temporality of KEs in a hypothesized MOA (Meek *et al.*, 2014). It should be appreciated that most MOA frameworks focus on carcinogenicity and thus dose and temporal concordance can be more easily assessed due to the usual delay in the onset of most tumors. However, because tissues constantly maintain homeostasis one KE does not necessarily occur to completion before the next KE begins. As such, early KEs tend to occur concomitantly. For example, chemical induced intestinal cytotoxicity does not necessarily result in obvious atrophy before regenerative processed begin (Bhat *et al.*, 2020; Cohen *et al.*, 2010; Cullen *et al.*, 2016). Table 2 shows the dose and temporal concordance of the KEs in the proposed MOA for liver changes following exposure to HFPO-DA. Regarding dose-response concordance, there is little evidence for transcriptomic, enzymatic (eg, β-oxidation), or histopathological changes in the mouse liver at 0.1 mg/kg after either 28 or 90 days of exposure, suggesting a potential threshold in response. Beginning at 0.5 mg/kg, there are transcriptomic responses related to PPAR signaling and fatty acid metabolism. At the tissue level, there is also evidence of hepatocellular hypertrophy in increased liver weight at ≥0.5 mg/kg. There is also evidence for PPARα activation, hepatocellular hypertrophy and hepatomegaly at 1 mg/kg. At 3 mg/kg, there is a large and statistically significant increase in β-oxidation activity. At 5 mg/kg, there is transcriptomic evidence for altered cell growth pathways related to mitotic and apoptotic signaling. At 5 mg/kg, there is also histopathological evidence for both mitosis and apoptosis.

**Table 2.** Dose and temporal concordance

	Temporal →					
	KE1. PPARα Activation		KE2. Alteration of Cell Growth Pathways		KE3. Perturbation of Cell Growth and Survival	
Dose (mg/kg)	28 days	90 days	28 days	90 days	28 days	90 days
0.1	No Δ in β-ox, No histopath effects^a^	No transcriptomic response, No histopath effects^c^^,^^d^	ND	No transcriptomic response^c^^,^^d^	No histopath effects^a^	No histopath effects^e^^,^^f^^,^^g^
0.5		↑PPARα signaling, ↑Peroxisomal lipid metabolism signaling, Hepatocellular hypertrophy^c–g^		No Δ in cell cycle signaling^c^^,^^d^		Hepatocellular hypertrophy, hepatomegaly^e^^,^^f^^,^^g^
1	↑PPARα signaling, Hepatocellular hypertrophy^b^		ND		Hepatomegaly^b^	
3	↑β-oxidation, Hepatocellular hypertrophy^a^		↑ Mitotic cell cycle signaling^i^^,^^j^		Hepatocellular hypertrophy, SSN^a^	
5		↑PPARα signaling, ↑Mitochondrial biogenesis signaling, hepatocellular hypertrophy^c–g^		↑ mitotic cell cycle signaling, ↑apoptotic signaling^c^^,^^d^		Mitosis, apoptosis, hepatomegaly, Hepatocellular hypertrophy^c^^,^^e^^,^^f^^,^^g^^,^^i^
30	↑β-oxidation, hepatocellular hypertrophy^a^		ND		Hepatocellular hypertrophy, mitosis, SSN^a^	

ND, no data available; SSN, single-cell necrosis and/or apoptosis.

a Haas (2008).

b Wang *et al.* (2017).

c Chappell *et al.* (2020).

d Heintz *et al.* (2022).

e DuPont (2010a).

f DuPont (2010b).

g NTP (2019).

h Guo *et al.* (2022).

i Thompson *et al*. (forthcoming).

j 2 mg/kg HFPO-DA.

The fourth evolved Bradford Hill consideration, consistency, asks whether the pattern of observations across species/strain/organs/test systems is what would be expected based on the hypothesized MOA (Meek *et al.*, 2014). In other MOA frameworks (eg, USEPA, 2005; Sonich-Mullin *et al.*, 2001) consistency has typically referred to the reproducibility of results across studies. Regarding the former, the tumor triad present in rats chronically exposed to HFPO-DA is similar to those observed with other PPARα activators. Regarding the latter definitions of consistency, multiple *in vivo* studies have demonstrated PPARα activation at the transcriptomic level (Chappell *et al.*, 2020; Guo *et al.*, 2022; Heintz *et al.*, 2022; Wang *et al.*, 2017), and multiple studies have reported similar histopathology (see Thompson *et al*., forthcoming).

The final evolved Bradford Hill consideration, analogy, questions whether the MOA would be anticipated based on broader chemical specific knowledge or information on chemically similar substances (Meek *et al.*, 2014). Indeed, legacy PFAS such as PFOA are known to activate PPARα along with mitigation of many effects in PPARα-null mice. As such, one would anticipate that many PFAS might have activity toward PPARα. The data herein clearly demonstrate that HFPO-DA shares PPARα activation with many other PFAS.

Regarding temporal concordance, there is molecular evidence for PPARα signaling at 28 days (the earliest timepoint with transcriptomic data yet investigated; Table 2). There is also evidence for hepatocellular hypertrophy, hepatomegaly, and mitosis at 28 days of exposure. One potential inconsistency in Table 2 is evidence for single cell necrosis/apoptosis (see discussion in Cytotoxicity section below) at 3 mg/kg HFPO-DA without reported evidence of mitotic figures at that dose at day 28. However, enrichment of cell cycle and DNA replication pathways was reported in mice exposed to various PFAS including HFPO-DA (2 mg/kg) for 28 days.

## Alternative MOAs for HFPO-DA-mediated liver effects in mice

The data show strong support for HFPO-DA functioning through the PPARα MOA in the mouse liver as described above. However, alternative MOAs have been hypothesized (USEPA, 2021), including cytotoxicity, mitochondrial dysfunction, and involvement of other PPARα subtypes. Each of these is addressed below.

### Cytotoxicity

Based on the evidence of PPARα activation and hepatomegaly observed in subchronic mouse studies, it is likely that mice would develop liver tumors under chronic HFPO-DA exposure scenarios. In a 2016 Toxicology Forum workshop on the human relevance of rodent liver tumors, 3 nongenotoxic MOAs (PPARα, constitutive androstane receptor [CAR], cytotoxicity) were discussed for their relevance for human health risk assessment (Felter *et al.*, 2018). The consensus, although not unanimous, was that PPARα- and CAR-mediated liver tumors were not relevant to humans, whereas cytotoxicity and regenerative hyperplasia were considered relevant to humans (Felter *et al.*, 2018). Among the six criteria listed for establishing a cytotoxic MOA were “clear evidence of cytotoxicity by histopathology, such as presence of necrosis and/or increased apoptosis”, “evidence of toxicity by increased serum enzymes that are relevant to humans”, and “presence of increased cell proliferation as evidenced by increased labeling index and/or increased number of hepatocytes” (Felter *et al.*, 2018). A fourth criteria, “the chemical is not DNA reactive”, explicitly considers evidence for an alternative MOA (ie, genotoxicity). The latter criterion, “not DNA reactive” comes from a data stream that has little in common with the other criteria that are markers of cytotoxicity. Clearly, other data streams are used for informing a cytotoxic MOA, and we believe that evidence streams supporting PPARα (as described above) should similarly factor into cytotoxic MOA determinations. As such, any toxicity that occurs as a result of PPARα mediated effects should not be interpreted as evidence for a cytotoxic MOA.

Regarding necrosis, the *NTP Nonneoplastic Lesion Atlas* states that various forms of necrosis (centrilobular, coagulation, focal, etc.) should not be subclassified with the exception of single cell necrosis. As such, it is unclear if single cell necrosis truly represents “necrosis” that is indicative of a cytotoxic MOA. As already discussed, there is evidence that some of the hepatocytes that might be considered necrotic stain positive for activated caspase-3 (Thompson *et al*., forthcoming). Notably, the only other form of necrosis that has been diagnosed in liver from HFPO-DA exposed mice is focal necrosis, which was not significantly elevated in exposed mice. Focal necrosis can occur in enlarged livers due to compression against the capsule or adjacent organs, resulting in focal hypoxia and cell death because the blood supply is already limited just below the capsule (Thoolen *et al.*, 2010). As discussed above, Felter *et al.* (2018) also include apoptosis and increased cell proliferation as evidence of a cytotoxic MOA; however, we have already discussed how these are known to occur in the PPARα MOA.

Recently, a gene expression signature indicative of liver cytotoxicity has been developed from short-term rat toxicity studies (Glaab *et al.*, 2021). Using published transcriptomic data (Chappell *et al.*, 2020; Heintz *et al.*, 2022), the expression of the 10 genes in the cytotoxicity gene set (CGS) were assessed in livers of mice exposed to HFPO-DA. Compared to PPARα-regulated genes involved in lipid transport and metabolism, which were largely induced at dose levels as low as 0.1 and 0.5 mg/kg HFPO-DA, these 10 genes associated/predictive of hepatic cytotoxicity were not significantly differentially expressed in either study, dose group or sex, with the exception of 4 genes (*Anxa2*, *Gpnmb*, *Timp1*, and *Tnfrsf12a*) in the highest dose group (5 mg/kg HFPO-DA) in parental male mice from the reproductive/developmental toxicity study (Figure 1C). That these 4 genes were elevated after the activation of PPARα pathways suggest they might be elevated as a result of PPARα activation. Moreover, the consistent evidence for increased PPARα-related gene expression in the absence of any change in the CGS in the female mice from the reproductive/developmental toxicity and male and female mice from the 90-day study support a PPARα MOA for the liver changes. The role(s) of each of these genes in cell physiology and toxicity are not fully known, and some of the gene changes might be downstream of PPARα activation. For example, glycoprotein nonmetastatic melanoma protein B (*Gpnmb*) appears to be a hepatokine that is secreted by the liver to promote lipogenesis in white adipose tissue (Gong *et al.*, 2019) and therefore might be related to PPARα-induced changes in lipid metabolism. Tumor necrosis factor receptor (*Tnfrsf12a*) codes for Fn14 that binds to tumor necrosis factor-like weak inducer of apoptosis (TWEAK) (Ratajczak *et al.*, 2022), which is potentially related to increased apoptosis present at 5 mg/kg. However, apoptosis was also observed at 5 mg/kg in other mice exposed to HFPO-DA that did not exhibit an increase in this gene (Figure 1C). Increased *Anxa2* expression has been observed in wild type but not humanized-PPARα mice treated with the PPARα-specific ligand WY-14643 (Yang *et al.*, 2008), suggesting species-specific PPARα-mediated gene expression of *Anxa2*. In addition, both *Timp1* and *Anxa2* may have roles in fibrosis (Wang *et al.*, 2022a; Zisser *et al.*, 2021); however, no histopathological evidence of fibrosis has been observed in tissue sections from the same mice the transcriptomic analyses were conducted. Overall, there is little/no transcriptomic evidence that HFPO-DA is acting via a cytotoxic MOA. Moreover, among various forms of programmed cell death (necroptosis, ferroptosis, pyroptosis, autophagy, and apoptosis), only apoptosis and autophagy gene sets were enriched in these transcriptomic studies (see Table 4 in Thompson *et al*., forthcoming)—both of which have been linked to PPARα (Byun *et al.*, 2020; Corton *et al.*, 2014; Xiao *et al.*, 2006).

In addition, mice exposed to 5 mg/kg HFPO-DA exhibit increased serum liver enzymes including alanine aminotransferase (ALT), aspartate aminotransferase (AST), alkaline phosphatase (ALP), and total bile acids. These changes in enzyme levels have been suggested to be indicative of a cytotoxic MOA (USEPA, 2021) based, in part, on criteria described in Hall *et al.*, (2012). Importantly in their 2012 review, Hall and colleagues concluded that rodent liver hypertrophy might be considered nonadverse for human health risk assessment when there is mechanistic evidence for nuclear receptor activation (eg, CAR, PXR, and PPARα) in the absence of other histological effects considered adverse such as necrosis, steatosis, fibrosis and cholestasis or large increases in serum liver enzymes such as ALT, AST, ALP, bile acids, and others. Serum liver enzyme levels in HFPO-DA exposed mice from the highest dose group (5 mg/kg) were increased greater than 2-fold compared to controls, exceeding what is considered nonadverse metabolic induction (Hall *et al.*, 2012), and suggesting potential for cytotoxicity. However, hepatic PPARα and the farnesoid X receptor (FXR) are key regulators of bile acid homeostasis, and PPARα activators and PFAS have been found to disrupt bile acid homeostasis in mice by altering gene expression of hepatic bile acid transporters and concurrently increasing serum liver enzyme levels in a PPARα-dependent manner (Cheng and Klaassen 2008; Gyamfi and Wan 2009; Liu *et al.*, 2014; Xie *et al.*, 2019). Perturbation of hepatic bile acid transporter gene expression, including the downregulation of the basolateral transporters, *Slc10a* (Na^+^-dependent bile acid transporter*, Ntcp*) and *Slco1a* (organic anion transporting polypeptide 1a1, *Oatp1a1*), also occurred in mice exposed to HFPO-DA (Figure 1C) (Chappell *et al.*, 2020; Guo *et al.*, 2022; Heintz *et al.*, 2022). It is conceivable that these PPARα-mediated changes in bile acids might have contributed to the gene changes within the CGS described above (Xie *et al.*, 2019). As a result, these effects are most likely part of a PPARα-mediated response in rodents and not indicative of non-PPARα mechanism that might be considered adverse in the context of human health risk assessment. These findings suggest that the criteria for assessing human relevant adverse effects in Hall *et al.* (2012) requires revisiting.

In addition to bile acid-associated increases in serum liver enzymes, it has also been demonstrated by partial hepatectomy-induced liver regeneration in rats, that liver cell proliferation also increases serum levels of liver enzymes, and these elevated serum enzyme levels are unrelated to hepatocellular necrosis and mitochondrial dysfunction (Contreras-Zentella and Hernández-Muñoz 2016; Díaz-Juárez *et al.*, 2006). Thus, liver enzyme release in mice treated with HFPO-DA may occur as a result of PPARα-related increases in enzyme expression and/or hepatic proliferation and not only as a consequence of necrotic cell death.

Toxicity studies for other PFAS, including PFNA and PFDA, observed increased serum liver enzymes in wild-type but not PPARα-null mice (Luo *et al.*, 2017; Zhang *et al.*, 2018). Administration of PPARα-specific ligands such as WY-14643, pemafibrate or fenofibrate has also been shown to increase serum liver enzymes in wild-type mice (Araki *et al.*, 2018; Gyamfi and Wan 2009). Therefore, the observed increase in ALT, AST, and ALP in mice exposed to HFPO-DA is likely PPARα-dependent.

### Other PPAR subtypes

Findings from 2 *in vitro* studies have been suggested to indicate a role for PPARγ in the MOA for HFPO-DA (USEPA, 2021). Li *et al.*, (2019) measured the transcriptional activation of mouse PPARγ by HFPO-DA in a reporter gene assay and determined that HFPO-DA weakly activated mouse PPARγ, with only a 1.2-fold increase in activity at the highest concentration tested. These authors also examined the binding affinity of HFPO-DA to the PPARγ ligand binding domain (LBD) and showed that HFPO-DA had little to no binding affinity to the mouse PPARγ LBD, with the IC_50_ value for HFPO-DA being indeterminate due to the minimal binding in the concentration range tested (up to 1 mM). Additionally, HFPO-DA caused minimal changes in PPARγ genes involved in adipogenesis in mouse adipocytes (3T3-L1 cells) (Li *et al.*, 2019). Evans *et al.* (2022) measured the transcriptional activation of rat PPARγ by HFPO-DA in a reporter gene assay and determined the lowest observed effect concentration (LOEC) producing a 2-fold increase over vehicle control for HFPO-DA and the ammonium salt of HFPO-DA was equal to 300 µM, the second highest concentration tested. It should be appreciated that the N-terminal binding domain of the PPARγ in this assay is substituted with the N-terminal binding domain of yeast GAL4, and that the luciferase is not being activated by a PPAR response element but rather the GAL4 upstream activator sequence (INDIGO Biosciences, State College, Pennsylvania). As such, these reporter gene assays should be interpreted with caution.

*In vivo* evidence for other PPAR subtypes is also limited and specific to the PPARγ subtype. Conley *et al.*, (2019; 2021) reported upregulation of several genes related to glucose metabolism in fetal and neonatal mouse livers exposed to HFPO-DA *in utero* (eg, *Pck1, Gk*, and *Aqp7*), that are associated with PPARγ signaling in adipose tissue (Rodríguez *et al.*, 2006); however, these gluconeogenesis genes are also regulated by PPARα in the liver (Kersten, 2014; Peeters and Baes, 2010). Therefore, it is likely that these genes were induced by PPARα rather than PPARγ based on increased expression of numerous other PPARα-regulated genes in maternal, fetal and neonatal livers (Conley *et al.*, 2019, 2021). As described in the MOA analysis above (see Supplementary Figure 1; Supplementary Table 1), HFPO-DA has little to no effect on the regulation of downstream genes or pathways associated with PPARγ (Chappell *et al.*, 2020; Heintz *et al.*, 2022). It should also be appreciated that there is very little expression of PPARγ in the rodent liver (Corrales *et al.*, 2018). As such, it is highly unlikely that PPARγ plays an important role in the MOA for HFPO-DA-induced liver changes in the mouse liver.

In addition to PPARγ, gene expression and pathway enrichment analysis of hepatic transcriptomic data from 90-day subchronic and reproductive/developmental toxicity studies for HFPO-DA in mice indicate little to no evidence for PPARδ activation (Supplementary Figure 1) (Chappell *et al.*, 2020; Heintz *et al.*, 2022). Although gene sets specific to PPARδ are not currently available within the canonical pathway subcollection used for gene set enrichment analyses in Chappell *et al.*, (2020) and Heintz *et al.*, (2022), individual genes associated with PPARδ signaling were investigated (Supplementary Figure 1). In addition, PPARδ and PPARγ are predominately expressed in muscle (Holst *et al.*, 2003) and adipose tissues (Chawla *et al.*, 1994), respectively, whereas PPARα is predominantly expressed in the liver (Corrales *et al.*, 2018). Overall, these data demonstrate that the weight of evidence for involvement of other PPAR subtypes in the MOA for HFPO-DA is poorly supported.

In contrast to HFPO-DA, studies on other PFAS examining the activation of PPARα compared to other nuclear receptors have reported mixed findings, with both PPARα-dependent and -independent effects. For example, Rosen *et al.*, (2017) reported upregulation of genes related to the activation of PPARγ and CAR, as well as PPARα in the livers of mice exposed to PFHxS or PFNA. These study authors also compared mouse liver gene expression data for PFOA and PFOS and concluded that over ∼75% of all genes regulated by these PFAS in wild-type mice are in fact PPARα-dependent (Rosen *et al.*, 2017). However, the activation of other nuclear receptors besides PPARα, and the extent to which they are activated, appears to be specific to individual PFAS and the model species being tested. As described in previous sections for rodents and in the sections below for humans, data for HFPO-DA clearly indicate that the compound is a PPARα-specific agonist.

### Mitochondrial dysfunction

Mitochondrial dysfunction as an alternative MOA for HFPO-DA-induced liver changes has been proposed (USEPA, 2021) based on reports of increased numbers of mitochondria in livers of mice exposed to HFPO-DA (Blake *et al.*, 2020) and increased hepatic expression of genes associated with mitochondrial β-oxidation in livers of mice and rats exposed to HFPO-DA (Chappell *et al.*, 2020; Conley *et al.*, 2021; Conley *et al.*, 2019; Heintz *et al.*, 2022). However, both peroxisomes and mitochondria play a critical role in lipid catabolism via β-oxidation of fatty acids, with each organelle metabolizing long-chain fatty acids or very long-chain fatty acids, respectively (Demarquoy and Le Borgne 2015; Tahri-Joutey *et al.*, 2021). Furthermore, some genes involved in mitochondrial β-oxidation are regulated by PPARα activators (Kersten and Stienstra 2017; Tahri-Joutey *et al.*, 2021). Aoyama *et al.*, (1998) showed that PPARα modulates the expression of genes involved in mitochondrial β-oxidation, as both peroxisomal and mitochondrial enzymes were induced following treatment with WY-14643 in wild type but not PPARα-null mice (Aoyama *et al.*, 1998). Similar findings have also been observed in mice treated with other PPARα agonists such as ciprofibrate (Cook *et al.*, 2000). In addition to PPARα, it is recognized the activity and abundance (ie, biogenesis) of peroxisomes and mitochondria are co-regulated in a PPARα- and PPARγ coactivator 1-alpha (PGC1α)-dependent manner (Fransen *et al.*, 2017). Transcriptomic analyses of livers from HFPO-DA-exposed mice indicate induction of both mitochondrial and peroxisomal fatty acid metabolism at similarly low median BMDs (ie, between 0.2–0.3 mg/kg for male mice and 0.5–0.9 mg/kg for female mice), and enrichment of gene sets related to mitochondrial biogenesis generally at higher BMDs (ie, median BMDs between 1.5 and 2.5 mg/kg; Figure 1B) (Chappell *et al.*, 2020; Heintz *et al.*, 2022). These data support PPARα’s role in maintaining systemic and cellular energy homeostasis by modulating the expression of genes involved in fatty acid β-oxidation and biogenesis for both peroxisomes and mitochondria (Aoyama *et al.*, 1998; Fransen *et al.*, 2017).

## Data gaps

A current data gap in the PPARα MOA is the absence of any *in vivo* studies examining the effects of HFPO-DA in PPARα-null mice. As indicated elsewhere in this article, the KEs described herein for HFPO-DA are often not observed when PPARα-null mice are exposed to classic PPARα activators such as WY or fibrates. We are currently in the process of conducting short-term assays in wild type and PPARα-null mice exposed to HFPO-DA, using exposure paradigms similar to those proposed for screening PFAS compounds (Gwinn *et al.*, 2020). Similarly, *in vitro* assays in wild type and PPARα-null mouse hepatocytes cells as well as human hepatocytes exposed to HFPO-DA can inform both the MOA and human relevance.

## Human relevance

It is widely accepted that rodent liver tumors resulting from exposure to PPARα activators are not relevant to humans (Corton *et al.*, 2018). While PPARα is expressed in many species and plays a role in lipid metabolism across species, the downstream cell proliferation signaling occurs specifically in rodents. Increased cell proliferation is a key and required event in the formation of rodent-specific hepatic tumors (Corton *et al.*, 2014). Yet, a remaining critical question is whether the non-neoplastic changes in the liver that occur during KE 2 and KE 3, as seen with PPARα activators like HFPO-DA, are unique to rodents.

The human relevance of the first 3 KEs underlying the PPARα MOA is summarized in Table 3. Experimental data for HFPO-DA exposure using human *in vitro* models are only available for KE 1; however, data are available for other PPARα activators that address the human relevance of KE 2 and KE 3. As shown in Table 3, only KE 1, PPARα activation, is shared across humans and rodents. Furthermore, within KE 1, only receptor activation and induction of lipid metabolism genes occur in both species.

**Table 3.** Human relevance of key events 1–3 within the PPARα MOA for HFPO-DA or PPARα-specific ligands

Key Event 1: PPARα Activation					Key Event 2: Alteration of Cell Growth Pathways					Key Event 3: Perturbation of Cell Growth and Survival				
Effects	**Result** ^a^	Compound(s)	Test System	References	Effect	**Result** ^a^	Compound(s)	Test System	References	Effect	**Result** ^a^	Compound(s)	Test System	References
PPARα receptor activation	**+**	HFPO-DA	Human PPARα reporter assay	Nielsen *et al.* (2022)	Increased DNA synthesis	**−**	Clofibric acid Bezafibrate Ciprofibrate Nafenopin	Primary human hepatocytes	Goll *et al.* (1999)	Increased liver size	**−**	Fenofibrate	Liver from hyperlipidemic patients	Gariot *et al.* (1987)
		HFPO-DA		Evans *et al.* (2022)			Clofibric acid Ciprofibrate		Perrone *et al.* (1998)	Increased serum liver enzymes^b^	**−**	Fenofibrate	Serum from type 2 diabetes mellitus patients	Black *et al.* (2014)
		HFPO-DA GW7647		Behr *et al.* (2020)			Clofibric acid		Elcombe *et al.* (1996)			Clofibrate	Serum from NAFLD or NASH patients	Laurin *et al.* (1996)
Lipid metabolism gene expression	**+**	HFPO-DA GW7647 WY-14643	HepG2 cells	Behr *et al.* (2020)	Cell proliferation signaling	**−**	GW7647		McMullen *et al.* (2014, 2020)			Fenofibrate		Fernández-Miranda *et al.* (2008)
		WY-14643	Primary human hepatocytes	Rakhshandehroo *et al.* (2009)	Pro-apoptotic regulation	**−**	GW7647		McMullen *et al.* (2014)			Gemfibrozil		Basaranoglu *et al.* (1999)
		GW7647		McMullen *et al.* (2014, 2020)										
Peroxisome proliferation	**−**	Clofibric acid Bezafibrate Ciprofibrate Nafenopin	Primary human hepatocytes	Goll *et al.* (1999)										
		Fenofibrate	Liver biopsies from hyperlipidemic patients	Gariot *et al.* (1987)										
		Gemfibrozil		De La Iglesia *et al.* (1982)										
		Fenofibrate		Blümcke *et al.* (1983)										

NAFLD, nonalcoholic fatty liver disease; NASH, nonalcoholic steatohepatitis.

a + indicates positive findings and − indicates negative findings in humans for an effect following exposure/treatment to the compound(s) listed.

b Putative marker of hepatocellular proliferation and PPARα-mediated bile acid perturbation in rodents.

Nielsen *et al.* (2022) determined that HFPO-DA acted as full human PPARα agonist using a full length human PPARα construct in a transactivation assay, with a potency (50% effective concentration) equal to 2.1 µM and an efficacy (maximum PPARα activity) of 134% compared to positive control levels. Evans *et al.* (2022) and Behr *et al.* (2020) observed similar results for HFPO-DA compared to other PFAS, as HFPO-DA had the greatest potency for human PPARα activation using a reporter assay construct consisting of human PPARα ligand binding domain fused with a Gal4 DNA-binding domain. Activation of PPARα by other PPARα-specific ligands and hyperlipidemic agents was also investigated by Evans *et al.* (2022), who reported similar 20% effective concentration (EC_20_) and area under the curve (AUC) values for HFPO-DA and clofibric acid (the metabolically active form of clofibrate) (Evans *et al.*, 2022). Consequently, at the same internal dose, clofibric acid and HFPO-DA may be expected to generate a similar level of PPARα activation in humans. However, the human effective clinical dose of fibrates, for example, TRICOR fenofibrate, is 0.69–2.1 mg/kg-day (assuming 70 kg adult) (USFDA, 2018), whereas RfD values for HFPO-DA are ≤0.01 mg/kg-day (Thompson *et al.*, 2019; USEPA, 2021).

*In vitro* studies in HepG2 or primary human hepatocytes treated with HFPO-DA or PPARα-specific ligands (ie, WY-14643 and GW7647), respectively, found induction of gene expression related to peroxisomal and mitochondrial fatty acid β-oxidation, lipid transport, and lipoprotein metabolism (Behr *et al.*, 2020; McMullen *et al.*, 2020; Rakhshandehroo *et al.*, 2009). The upregulation of hepatic lipid metabolism functional categories in primary hepatocytes was well-conserved between humans and mice (Rakhshandehroo *et al.*, 2009) or rats (McMullen *et al.*, 2020). However, at similar treatment concentrations, these conserved gene expression changes between species occurred at lower expression levels in humans compared to rodents, resulting in an overall lower transcriptional response to PPARα activation in humans (Bjork *et al.*, 2011; McMullen *et al.*, 2020; Rakhshandehroo *et al.*, 2009). In addition, induction of hepatic peroxisome proliferation by PPARα activators is not conserved across species. Bentley *et al.* (1993) reviewed the significance of hepatic peroxisome proliferation in humans and concluded that the available data from hypolipidemic patient biopsies demonstrated an overall absence of increased peroxisome proliferation in the livers of patients treated with hypolipidemic drugs (eg, fenofibrate and gemfibrozil). Goll *et al.*, (1999) also confirmed a lack of peroxisome proliferation-associated parameters in human hepatocyte cultures treated with various hypolipidemic agents (Table 3).

Evidence for KE 2, alteration of cell growth pathways, is absent in primary human hepatocytes treated with PPARα-specific ligands or hypolipidemic drugs, with no indication of increased DNA synthesis (Elcombe *et al.*, 1996; Goll *et al.*, 1999; Perrone *et al.*, 1998), enrichment of cellular pathways related to cell proliferation, or induction of pro-apoptotic gene expression (Table 3) (McMullen *et al.*, 2020; McMullen *et al.*, 2014). Based on hepatic gene expression results *in vitro* for PPARα-specific ligand WY-14643, Rakhshandehroo *et al.*, (2009) concluded that “PPARα regulates a mostly (ie, besides lipid modulating effects) divergent set of genes in mouse and human liver”. In addition, the human data available for KE 3, perturbation of cell growth and survival, show that liver size and serum liver enzyme levels (a putative marker of hepatocellular proliferation and PPARα-mediated bile acid perturbation in rodents), do not increase in patients treated with hypolipidemic drugs (Table 3) (Black *et al.*, 2014; Gariot *et al.*, 1987; Liss and Finck 2017). Therefore, the human evidence base for HFPO-DA and other potent PPARα agonists demonstrates that the non-neoplastic changes (ie, KE 2 and 3) observed in the livers of mice treated with HFPO-DA do not occur in humans.

## Discussion

The weight of evidence from mechanistic and phenotypic data described herein strongly supports that the liver changes observed in mice exposed to HFPO-DA are occurring via a PPARα MOA. The central role of PPARα in the observed effects in mouse livers is summarized in Figure 1D. The relevance of PPARα-related liver toxicity in human health risk assessment has been a topic of great interest. In a review on the adversity of liver hypertrophy, experts concluded that “hepatomegaly as a consequence of hepatocellular hypertrophy without histologic or clinical pathology alterations indicative of liver toxicity was considered an adaptive and a nonadverse reaction” (Hall *et al.*, 2012). Therein, evidence for PPARα and CAR activation in the absence of hepatoxicity was considered nonadverse. Being somewhat dated, the language on liver necrosis was somewhat vague and it was not considered that some changes in serum liver enzymes could be a direct consequence of PPARα-mediated gene changes as opposed to cytotoxicity. Nevertheless, there was a general view that PPARα and CAR mediated liver changes were “rodent-specific phenomenon.”

The USEPA (2021) risk assessment of HFPO-DA indicates that a PPARα MOA would potentially have limited relevance for humans:

“The increases in relative liver weight, hepatocellular hypertrophy, and peroxisome activity (eg, peroxisomal beta-oxidation induction) can be associated with activation of cellular peroxisome proliferator-activated receptor alpha (PPARα) receptors, making it difficult to determine if this change is a reflection of PPARα activation or an indication of GenX chemical toxicity. This is important because the PPARα response could be more relevant to rodents than humans.”

Rather than directly addressing human relevance, the USEPA (2021) instead hypothesized several alternative MOAs for HFPO-DA-induced liver effects. For the reference dose (RfD) derivation, the USEPA modeled the combined incidence of several different liver lesions (hepatocellular hypertrophy/cytoplasmic alteration, single cell necrosis, apoptosis, and focal necrosis). Available scientific evidence strongly supports that these liver lesions in mice occur as part of the PPARα MOA and therefore are not relevant to humans. In contrast, the evidence for alternative MOAs for HFPO-DA-induced liver toxicity is not supported by the scientific literature. As such, these liver endpoints should not be used as the point of departure (POD) in developing a RfD for HFPO-DA. Additionally, given that data in the scientific literature demonstrate that other PFAS are PPARα agonists (Evans *et al.*, 2022; Nielsen *et al.*, 2022), candidate toxicity values or relative potency factors (RPFs) for other PFAS derived from liver endpoints in rodents should also be evaluated in the context of this MOA.

Additionally, in developing the proposed RfD, the USEPA applied a 3-fold interspecies uncertainty factor after accounting for potential interspecies pharmacokinetic differences via allometric scaling. This 3-fold adjustment is not necessary, as the considerable evidence that the liver lesions have limited human relevance indicate that humans are unlikely to be more sensitive to HFPO-DA than mice. In addition to a 10-fold uncertainty factor for database uncertainty and a 10-fold factor for human variability, USEPA also applied a full 10-fold uncertainty factor for use of a subchronic study. In totality, USEPA’s maximum 3000-fold uncertainty factor was applied to a rodent specific endpoint, resulting in an RfD of 0.000003 mg/kg-day, one of the lowest RfD values in the IRIS database.

A series of recent papers provides a means of ground-truthing the RfD developed for HFPO-DA. Although the diversity of PFAS warrant chemical specific assessments, a recent article demonstrated the potential application of the concept of threshold for toxicological concern (TTC) to PFAS (Lea *et al.*, 2022). Therein, data for 27 PFAS were classified via ToxTree (Patlewicz *et al.*, 2008) as Cramer Class III structures (Cramer Class III chemical structures contain “elements other than carbon, hydrogen, oxygen, nitrogen or divalent sulfur” with structural features that “permit no strong initial presumption of safety or may even suggest significant toxicity” (Cramer *et al.*, 1978), which have a current fifth percentile no-observed-adverse-effect level (NOAEL) value of 1.5 µg/kg/day. The expansion of the chemical space via the addition of PFAS resulted in a fifth percentile NOAEL value of 1.3 µg/kg-day (0.0013 mg/kg-day). An earlier evaluation demonstrated that, on average, Cramer Class III TTC values are ∼6-fold lower than corresponding IRIS values for the same chemical (Pham *et al.*, 2020), indicating that TTC values, which are generally used in data-poor situations, are more conservative than detailed risk assessment toxicity values (eg, RfDs). For HFPO-DA, the TTC value is 433-fold *higher* than the RfD. As noted in Pham *et al.* (2020), a cursory examination of chemicals where the TTC value is significantly higher than corresponding RfD value indicated potential need for reevaluation of the RfD due to outdated methodology or overapplication/compounding of uncertainty factors. The fact that the RfD for HFPO-DA is significantly lower than the Cramer Class III TTC provides an additional line of evidence indicating that the RfD for HFPO-DA is overly conservative. As indicated above, the application of a maximum 3000-fold uncertainty factor to a rodent specific endpoint warrants reexamination.

In conclusion, the current weight of evidence indicates that the HFPO-DA induced effects in the mouse liver are the result of a PPARα MOA. These effects are widely considered to have limited relevance to humans in the context of tumor formation (Corton *et al.*, 2018; Felter *et al.*, 2018). Hepatomegaly, and early precursor in the development of rodent liver tumors, is considered nonadverse when there is evidence for PPARα or CAR activation (Hall *et al.*, 2012). Data indicate that some effects traditionally considered as markers of hepatotoxicity are PPARα mediated and not necessarily indicative of liver toxicity. As such, the liver changes in mice following exposure to HFPO-DA are not relevant for human health risk assessment.

## Supplementary Material

kfad004_Supplementary_Data

## References

[kfad004-B1] Anderson J. K. , Brecher R. W., Cousins I. T., DeWitt J., Fiedler H., Kannan K., Kirman C. R., Lipscomb J., Priestly B., Schoeny R., et al (2022). Grouping of PFAS for human health risk assessment: Findings from an independent panel of experts. Regul. Toxicol. Pharmacol. 134, 105226. 35817206 10.1016/j.yrtph.2022.105226

[kfad004-B2] Aoyama T. , Peters J. M., Iritani N., Nakajima T., Furihata K., Hashimoto T., Gonzalez F. J. (1998). Altered constitutive expression of fatty acid-metabolizing enzymes in mice lacking the peroxisome proliferator-activated receptor alpha (PPARalpha). J. Biol. Chem. 273, 5678–5684. 9488698 10.1074/jbc.273.10.5678

[kfad004-B3] Araki M. , Nakagawa Y., Oishi A., Han S. I., Wang Y., Kumagai K., Ohno H., Mizunoe Y., Iwasaki H., Sekiya M., et al (2018). The peroxisome proliferator-activated receptor α (PPARα) agonist pemafibrate protects against diet-induced obesity in mice. Int. J. Mol. Sci. 19. 10.3390/ijms19072148 PMC6073532 30041488

[kfad004-B2971479] Basaranoglu M., , Acbay O., and , Sonsuz A. (1999). A controlled trial of gemfibrozil in the treatment of patients with nonalcoholic steatohepatitis. J. Hepatol. 31, 384. 10453959 10.1016/s0168-8278(99)80243-8

[kfad004-B4] Behr A. C. , Plinsch C., Braeuning A., Buhrke T. (2020). Activation of human nuclear receptors by perfluoroalkylated substances (PFAS). Toxicol. In Vitro 62, 104700. 31676336 10.1016/j.tiv.2019.104700

[kfad004-B5] Bentley P. , Calder I., Elcombe C., Grasso P., Stringer D., Wiegand H. J. (1993). Hepatic peroxisome proliferation in rodents and its significance for humans. Food Chem. Toxicol. 31, 857–907. 8258416 10.1016/0278-6915(93)90225-n

[kfad004-B6] Bhat V. S. , Cohen S. M., Gordon E. B., Wood C. E., Cullen J. M., Harris M. A., Proctor D. M., Thompson C. M. (2020). An adverse outcome pathway for small intestinal tumors in mice involving chronic cytotoxicity and regenerative hyperplasia: A case study with hexavalent chromium, captan, and folpet. Crit. Rev. Toxicol. 50, 685–706. 33146058 10.1080/10408444.2020.1823934

[kfad004-B7] Bjork J. A. , Butenhoff J. L., Wallace K. B. (2011). Multiplicity of nuclear receptor activation by PFOA and PFOS in primary human and rodent hepatocytes. Toxicology 288, 8–17. 21723365 10.1016/j.tox.2011.06.012

[kfad004-B8] Black R. N. , Ennis C. N., Young I. S., Hunter S. J., Atkinson A. B., Bell P. M. (2014). The peroxisome proliferator-activated receptor alpha agonist fenofibrate has no effect on insulin sensitivity compared to atorvastatin in type 2 diabetes mellitus; a randomised, double-blind controlled trial. J. Diabetes Complications 28, 323–327. 24560135 10.1016/j.jdiacomp.2014.01.001

[kfad004-B9] Blake B. E. , Cope H. A., Hall S. M., Keys R. D., Mahler B. W., McCord J., Scott B., Stapleton H. M., Strynar M. J., Elmore S. A., et al (2020). Evaluation of maternal, embryo, and placental effects in CD-1 mice following gestational exposure to perfluorooctanoic acid (PFOA) or hexafluoropropylene oxide dimer acid (HFPO-DA or GenX). Environ. Health Perspect. 128, 27006. 32074459 10.1289/EHP6233 PMC7064328

[kfad004-B10] Boskou D. , Blekas G., Tsimidou M. (2006). Olive oil composition. In *Olive Oil: Chemistry and Technology*, 2nd ed. (D. Boskou, Ed.), pp. 41–72. AOCS Press, Champaign, IL.

[kfad004-B9907522] Blümcke S., , Schwartzkopff W., , Lobeck H., , Edmondson N. A., , Prentice D. E., and , Blane G. F. (1983). Influence of fenofibrate on cellular and subcellular liver structure in hyperlipidemic patients. *Atherosclerosis* 46, 105–116. 10.1016/0021-9150(83)90169-7 6838687

[kfad004-B11] Byun S. , Seok S., Kim Y.-C., Zhang Y., Yau P., Iwamori N., Xu H. E., Ma J., Kemper B., Kemper J. K. (2020). Fasting-induced FGF21 signaling activates hepatic autophagy and lipid degradation via JMJD3 histone demethylase. Nat. Commun. 11, 807. 32042044 10.1038/s41467-020-14384-z PMC7010817

[kfad004-B12] Caverly Rae J. M. , Craig L., Slone T. W., Frame S. R., Buxton L. W., Kennedy G. L. (2015). Evaluation of chronic toxicity and carcinogenicity of ammonium 2,3,3,3-tetrafluoro-2-(heptafluoropropoxy)-propanoate in Sprague-Dawley rats. Toxicol. Rep. 2, 939–949. 28962433 10.1016/j.toxrep.2015.06.001 PMC5598527

[kfad004-B13] Chappell G. A. , Thompson C. M., Wolf J. C., Cullen J. M., Klaunig J. E., Haws L. C. (2020). Assessment of the mode of action underlying the effects of GenX in mouse liver and implications for assessing human health risks. Toxicol. Pathol. 48, 494–508. 32138627 10.1177/0192623320905803 PMC7153225

[kfad004-B14] Chawla A. , Schwarz E. J., Dimaculangan D. D., Lazar M. A. (1994). Peroxisome proliferator-activated receptor (PPAR) gamma: Adipose-predominant expression and induction early in adipocyte differentiation. Endocrinology 135, 798–800. 8033830 10.1210/endo.135.2.8033830

[kfad004-B15] Cheng X. , Klaassen C. D. (2008). Critical role of PPAR-alpha in perfluorooctanoic acid- and perfluorodecanoic acid-induced downregulation of Oatp uptake transporters in mouse livers. Toxicol. Sci. 106, 37–45. 18703564 10.1093/toxsci/kfn161 PMC2563139

[kfad004-B16] Cohen S. M. , Gordon E. B., Singh P., Arce G. T., Nyska A. (2010). Carcinogenic mode of action of folpet in mice and evaluation of its relevance to humans. Crit. Rev. Toxicol. 40, 531–545. 20521864 10.3109/10408441003742903

[kfad004-B17] Conley J. M. , Lambright C. S., Evans N., McCord J., Strynar M. J., Hill D., Medlock-Kakaley E., Wilson V. S., Gray L. E. Jr. (2021). Hexafluoropropylene oxide-dimer acid (HFPO-DA or GenX) alters maternal and fetal glucose and lipid metabolism and produces neonatal mortality, low birthweight, and hepatomegaly in the Sprague-Dawley rat. Environ. Int. 146, 106204. 33126064 10.1016/j.envint.2020.106204 PMC7775906

[kfad004-B18] Conley J. M. , Lambright C. S., Evans N., Strynar M. J., McCord J., McIntyre B. S., Travlos G. S., Cardon M. C., Medlock-Kakaley E., Hartig P. C., et al (2019). Adverse maternal, fetal, and postnatal effects of hexafluoropropylene oxide dimer acid (GenX) from oral gestational exposure in Sprague-Dawley rats. Environ. Health Perspect. 127, 37008. 30920876 10.1289/EHP4372 PMC6768323

[kfad004-B19] Contreras-Zentella M. L. , Hernández-Muñoz R. (2016). Is liver enzyme release really associated with cell necrosis induced by oxidant stress? Oxid. Med. Cell. Longev. 2016, 3529149. 26798419 10.1155/2016/3529149 PMC4699024

[kfad004-B20] Cook W. S. , Yeldandi A. V., Rao M. S., Hashimoto T., Reddy J. K. (2000). Less extrahepatic induction of fatty acid beta-oxidation enzymes by PPAR alpha. Biochem. Biophys. Res. Commun. 278, 250–257. 11071880 10.1006/bbrc.2000.3739

[kfad004-B21] Corrales P. , Vidal-Puig A., Medina-Gómez G. (2018). PPARs and metabolic disorders associated with challenged adipose tissue plasticity. Int. J. Mol. Sci. 19. 10.3390/ijms19072124 PMC6073677 30037087

[kfad004-B6042199] Corton J. C., , Hill T., , Sutherland J. J., , Stevens J. L., and , Rooney J. (2020). A set of six Gene expression biomarkers identify rat liver tumorigens in short-term assays. *Toxicol. Sc*i*. *177, 11–26. 10.1093/toxsci/kfaa101 PMC8026143 32603430

[kfad004-B22] Corton J. C. , Cunningham M. L., Hummer B. T., Lau C., Meek B., Peters J. M., Popp J. A., Rhomberg L., Seed J., Klaunig J. E. (2014). Mode of action framework analysis for receptor-mediated toxicity: The peroxisome proliferator-activated receptor alpha (PPARα) as a case study. Crit. Rev. Toxicol. 44, 1–49. 10.3109/10408444.2013.835784 24180432

[kfad004-B23] Corton J. C. , Peters J. M., Klaunig J. E. (2018). The PPARalpha-dependent rodent liver tumor response is not relevant to humans: Addressing misconceptions. Arch. Toxicol. 92, 83–119. 29197930 10.1007/s00204-017-2094-7 PMC6092738

[kfad004-B24] Costello E. , Rock S., Stratakis N., Eckel S. P., Walker D. I., Valvi D., Cserbik D., Jenkins T., Xanthakos S. A., Kohli R., et al (2022). Exposure to per- and polyfluoroalkyl substances and markers of liver injury: A systematic review and meta-analysis. Environ. Health Perspect. 130, 46001. 35475652 10.1289/EHP10092 PMC9044977

[kfad004-B9471664] Cramer G. M., , Ford R. A., and , Hall R. L. (1978). Estimation of toxic hazard-a decision tree approach. Food Cosmet. Toxicol. 16, 255–276. 357272 10.1016/s0015-6264(76)80522-6

[kfad004-B25] Cullen J. M. , Ward J. M., Thompson C. M. (2016). Reevaluation and classification of duodenal lesions in B6C3F1 mice and F344 rats from 4 studies of hexavalent chromium in drinking water. Toxicol. Pathol. 44, 279–289. 26538584 10.1177/0192623315611501 PMC4785997

[kfad004-B28102895] De La Iglesia F. A., , Lewis J. E., , Buchanan R. A., , Marcus E. L., and , McMahon G. (1982). Light and electron microscopy of liver in hyperlipoproteinemic patients under long-term gemfibrozil treatment. *Atherosclerosis* 43, 19–37. 10.1016/0021-9150(82)90096-x 6807326

[kfad004-B26] Demarquoy J. , Le Borgne F. (2015). Crosstalk between mitochondria and peroxisomes. World J. Biol. Chem. 6, 301–309. 26629313 10.4331/wjbc.v6.i4.301 PMC4657118

[kfad004-B27] Díaz-Juárez J. , Rivera-Valerdi L., Bernal-Cerrillo D. E., Hernández-Muñoz R. (2006). Predominance of released mitochondrial enzymes by partial hepatectomy-induced rat regenerating liver is controlled by hemodynamic changes and not related to mitochondrial damage. Scand. J. Gastroenterol. 41, 223–233. 16484128 10.1080/00365520510024142

[kfad004-B28] DuPont. (2010a). E.I. Du Pont De Nemours and Company. An oral (gavage) reproduction/developmental toxicity screening study of h-28548 in mice. U.S. EPA OPPTS 870.3550; OECD test guideline 421. Study conducted by WIL Research Laboratories, LLC (study completion date: December 29, 2010). Ashland, OH.

[kfad004-B29] DuPont. (2010b). E.I. Du pOnt De Nemours and Company. H-28548: Subchronic toxicity 90-day gavage study in mice. OECD test guideline 408. Study conducted by E.I. Du Pont De Nemours and Company Dupont Haskell Global Centers for Health & Environmental Sciences (study completion date: February 19, 2010).

[kfad004-B30] Elcombe C. R. , Bell D. R., Elias E., Hasmall S. C., Plant N. J. (1996). Peroxisome proliferators: Species differences in response of primary hepatocyte cultures. Ann. N. Y. Acad. Sci. 804, 628–635. 8993577 10.1111/j.1749-6632.1996.tb18649.x

[kfad004-B31] Evans N. , Conley J. M., Cardon M., Hartig P., Medlock-Kakaley E., Gray L. E. Jr. (2022). In vitro activity of a panel of per- and polyfluoroalkyl substances (PFAS), fatty acids, and pharmaceuticals in peroxisome proliferator-activated receptor (PPAR) alpha, PPAR gamma, and estrogen receptor assays. Toxicol. Appl. Pharmacol. 449, 116136. 35752307 10.1016/j.taap.2022.116136 PMC9341220

[kfad004-B32] Felter S. P. , Foreman J. E., Boobis A., Corton J. C., Doi A. M., Flowers L., Goodman J., Haber L. T., Jacobs A., Klaunig J. E., et al (2018). Human relevance of rodent liver tumors: Key insights from a toxicology forum workshop on nongenotoxic modes of action. Regul. Toxicol. Pharmacol. 92, 1–7. 29113941 10.1016/j.yrtph.2017.11.003 PMC11350555

[kfad004-B2804563] Fernández-Miranda C., , Pérez-Carreras M., , Colina F., , López-Alonso G., , Vargas C., and , Solís-Herruzo J. A. (2008). A pilot trial of fenofibrate for the treatment of non-alcoholic fatty liver disease. Dig. Liver Dis. 40, 200–205. 18261709 10.1016/j.dld.2007.10.002

[kfad004-B33] Fransen M. , Lismont C., Walton P. (2017). The peroxisome-mitochondria connection: How and why? Int. J. Mol. Sci. 18, 1126. 28538669 10.3390/ijms18061126 PMC5485950

[kfad004-B34] Gannon S. A. , Fasano W. J., Mawn M. P., Nabb D. L., Buck R. C., Buxton L. W., Jepson G. W., Frame S. R. (2016). Absorption, distribution, metabolism, excretion, and kinetics of 2,3,3,3-tetrafluoro-2-(heptafluoropropoxy)propanoic acid ammonium salt following a single dose in rat, mouse, and cynomolgus monkey. Toxicology 340, 1–9. 26743852 10.1016/j.tox.2015.12.006

[kfad004-B35] Gariot P. , Barrat E., Drouin P., Genton P., Pointel J. P., Foliguet B., Kolopp M., Debry G. (1987). Morphometric study of human hepatic cell modifications induced by fenofibrate. Metabolism 36, 203–210. 3821501 10.1016/0026-0495(87)90177-6

[kfad004-B36] Glaab W. E. , Holder D., He Y. D., Bailey W. J., Gerhold D. L., Beare C., Erdos Z., Lane P., Michna L., Muniappa N., et al (2021). Universal toxicity gene signatures for early identification of drug-induced tissue injuries in rats. Toxicol. Sci. 181, 148–159. 33837425 10.1093/toxsci/kfab038

[kfad004-B37] Goll V. , Alexandre E., Viollon-Abadie C., Nicod L., Jaeck D., Richert L. (1999). Comparison of the effects of various peroxisome proliferators on peroxisomal enzyme activities, DNA synthesis, and apoptosis in rat and human hepatocyte cultures. Toxicol. Appl. Pharmacol. 160, 21–32. 10502499 10.1006/taap.1999.8737

[kfad004-B38] Gong X. M. , Li Y. F., Luo J., Wang J. Q., Wei J., Wang J. Q., Xiao T., Xie C., Hong J., Ning G., et al (2019). Gpnmb secreted from liver promotes lipogenesis in white adipose tissue and aggravates obesity and insulin resistance. Nat. Metab. 1, 570–583. 32694855 10.1038/s42255-019-0065-4

[kfad004-B39] Guo H. , Chen J., Zhang H., Yao J., Sheng N., Li Q., Guo Y., Wu C., Xie W., Dai J. (2022). Exposure to GenX and its novel analogs disrupts hepatic bile acid metabolism in male mice. Environ. Sci. Technol. 56, 6133–6143. 34427428 10.1021/acs.est.1c02471

[kfad004-B40] Guo H. , Sheng N., Guo Y., Wu C., Xie W., Dai J. (2021). Exposure to GenX and its novel analogs disrupts fatty acid metabolism in male mice. Environ. Pollut. 291, 118202. 34562693 10.1016/j.envpol.2021.118202

[kfad004-B41] Gwinn W. M. , Auerbach S. S., Parham F., Stout M. D., Waidyanatha S., Mutlu E., Collins B., Paules R. S., Merrick B. A., Ferguson S., et al (2020). Evaluation of 5-day in vivo rat liver and kidney with high-throughput transcriptomics for estimating benchmark doses of apical outcomes. Toxicol. Sci. 176, 343–354. 32492150 10.1093/toxsci/kfaa081 PMC7416315

[kfad004-B42] Gyamfi M. A. , Wan Y. J. (2009). Mechanisms of resistance of hepatocyte retinoid x receptor alpha-null mice to WY-14,643-induced hepatocyte proliferation and cholestasis. J. Biol. Chem. 284, 9321–9330. 19176532 10.1074/jbc.M808861200 PMC2666584

[kfad004-B43] Haas M. C. (2008). A 28-Day Oral (Gavage) Toxicity Study of h-28397 in Mice with a 28-Day Recovery (Dupont). Ashland, OH: WIL Research Laboratories, LLC. https://hero.Epa.Gov/hero/index.Cfm/project/page/page/1/rows/10/sort/year%20desc/format/list/project_id/2627/criteria_all/dupont/usage_searchtype/any. Accessed: October 18, 2022.

[kfad004-B44] Hall A. P. , Elcombe C. R., Foster J. R., Harada T., Kaufmann W., Knippel A., Küttler K., Malarkey D. E., Maronpot R. R., Nishikawa A., et al (2012). Liver hypertrophy: A review of adaptive (adverse and non-adverse) changes–conclusions from the 3rd international ESTP expert workshop. Toxicol. Pathol. 40, 971–994. 22723046 10.1177/0192623312448935

[kfad004-B45] Hanahan D. , Weinberg R. A. (2011). Hallmarks of cancer: The next generation. Cell 144, 646–674. 21376230 10.1016/j.cell.2011.02.013

[kfad004-B46] Heintz M. M. , Chappell G. A., Thompson C. M., Haws L. C. (2022). Evaluation of transcriptomic responses in livers of mice exposed to the short-chain PFAS compound HFPO-DA. Front. Toxicol. 4, 937168. 35832492 10.3389/ftox.2022.937168 PMC9271854

[kfad004-B47] Hill A. B. (1965). The environment and disease: Association or causation? Proc. R. Soc. Med. 58, 295–300. 14283879 10.1177/003591576505800503 PMC1898525

[kfad004-B48] Holst D. , Luquet S., Nogueira V., Kristiansen K., Leverve X., Grimaldi P. A. (2003). Nutritional regulation and role of peroxisome proliferator-activated receptor delta in fatty acid catabolism in skeletal muscle. Biochim. Biophys. Acta 1633, 43–50. 12842194 10.1016/s1388-1981(03)00071-4

[kfad004-B49] Kersten S. (2014). Integrated physiology and systems biology of PPARα. Mol. Metab. 3, 354–371. 24944896 10.1016/j.molmet.2014.02.002 PMC4060217

[kfad004-B50] Kersten S. , Stienstra R. (2017). The role and regulation of the peroxisome proliferator activated receptor alpha in human liver. Biochimie 136, 75–84. 28077274 10.1016/j.biochi.2016.12.019

[kfad004-B51] Klaunig J. E. , Babich M. A., Baetcke K. P., Cook J. C., Corton J. C., David R. M., DeLuca J. G., Lai D. Y., McKee R. H., Peters J. M., et al (2003). PPARalpha agonist-induced rodent tumors: Modes of action and human relevance. Crit. Rev. Toxicol. 33, 655–780. 14727734 10.1080/713608372

[kfad004-B52] Klaunig J. E. , Hocevar B. A., Kamendulis L. M. (2012). Mode of action analysis of perfluorooctanoic acid (PFOA) tumorigenicity and human relevance. Reprod. Toxicol. 33, 410–418. 22120428 10.1016/j.reprotox.2011.10.014

[kfad004-B53] Lau C. , Anitole K., Hodes C., Lai D., Pfahles-Hutchens A., Seed J. (2007). Perfluoroalkyl acids: A review of monitoring and toxicological findings. Toxicol. Sci. 99, 366–394. 17519394 10.1093/toxsci/kfm128

[kfad004-B3208625] Laurin J., , Lindor K. D., , Crippin J. S., , Gossard A., , Gores G. J., , Ludwig J., , Rakela J., and , McGill D. B. (1996). Ursodeoxycholic acid or clofibrate in the treatment of non-alcohol-induced steatohepatitis: a pilot study. Hepatology 23, 1464–1467. 8675165 10.1002/hep.510230624

[kfad004-B54] Lea I. A. , Pham L. L., Antonijevic T., Thompson C., Borghoff S. J. (2022). Assessment of the applicability of the threshold of toxicological concern for per- and polyfluoroalkyl substances. Regul. Toxicol. Pharmacol. 133, 105190. 35662637 10.1016/j.yrtph.2022.105190

[kfad004-B55] Li C. H. , Ren X. M., Guo L. H. (2019). Adipogenic activity of oligomeric hexafluoropropylene oxide (perfluorooctanoic acid alternative) through peroxisome proliferator-activated receptor γ pathway. Environ. Sci. Technol. 53, 3287–3295. 30785727 10.1021/acs.est.8b06978

[kfad004-B56] Liss K. H. , Finck B. N. (2017). PPARS and nonalcoholic fatty liver disease. Biochimie 136, 65–74. 27916647 10.1016/j.biochi.2016.11.009 PMC5380579

[kfad004-B57] Liu A. , Krausz K. W., Fang Z. Z., Brocker C., Qu A., Gonzalez F. J. (2014). Gemfibrozil disrupts lysophosphatidylcholine and bile acid homeostasis via PPARα and its relevance to hepatotoxicity. Arch. Toxicol. 88, 983–996. 24385052 10.1007/s00204-013-1188-0 PMC6398607

[kfad004-B58] Luo M. , Tan Z., Dai M., Song D., Lin J., Xie M., Yang J., Sun L., Wei D., Zhao J., et al (2017). Dual action of peroxisome proliferator-activated receptor alpha in perfluorodecanoic acid-induced hepatotoxicity. Arch. Toxicol. 91, 897–907. 27344344 10.1007/s00204-016-1779-7 PMC6350782

[kfad004-B59] McMullen P. D. , Bhattacharya S., Woods C. G., Pendse S. N., McBride M. T., Soldatow V. Y., Deisenroth C., LeCluyse E. L., Clewell R. A., Andersen M. E. (2020). Identifying qualitative differences in PPARα signaling networks in human and rat hepatocytes and their significance for next generation chemical risk assessment methods. Toxicol. In Vitro 64, 104463. 31628012 10.1016/j.tiv.2019.02.017

[kfad004-B60] McMullen P. D. , Bhattacharya S., Woods C. G., Sun B., Yarborough K., Ross S. M., Miller M. E., McBride M. T., LeCluyse E. L., Clewell R. A., et al (2014). A map of the PPARα transcription regulatory network for primary human hepatocytes. Chem. Biol. Interact. 209, 14–24. 24269660 10.1016/j.cbi.2013.11.006

[kfad004-B61] Meek M. E. , Palermo C. M., Bachman A. N., North C. M., Jeffrey Lewis R. (2014). Mode of action human relevance (species concordance) framework: Evolution of the Bradford Hill considerations and comparative analysis of weight of evidence. J. Appl. Toxicol. 34, 595–606. 24777878 10.1002/jat.2984 PMC4321063

[kfad004-B62] Morimura K. , Cheung C., Ward J. M., Reddy J. K., Gonzalez F. J. (2006). Differential susceptibility of mice humanized for peroxisome proliferator-activated receptor alpha to WY-14,643-induced liver tumorigenesis. Carcinogenesis 27, 1074–1080. 16377806 10.1093/carcin/bgi329 PMC1447533

[kfad004-B63] Nielsen G. , Heiger-Bernays W. J., Schlezinger J. J., Webster T. F. (2022). Predicting the effects of per- and polyfluoroalkyl substance mixtures on peroxisome proliferator-activated receptor alpha activity in vitro. Toxicology 465, 153024. 34743024 10.1016/j.tox.2021.153024 PMC8692422

[kfad004-B64] NTP. (2019). National toxicology program. Final report on the pathology peer review of liver findings from h-28548: Subchronic toxicity 90-day gavage study in mice and an oral (gavage) reproduction/developmental toxicity screening study of h-28548 in mice. Hero id: 6985027.

[kfad004-B65] Peeters A. , Baes M. (2010). Role of PPARalpha in hepatic carbohydrate metabolism. PPAR Res. 2010, 1. 10.1155/2010/572405 PMC2948921 20936117

[kfad004-B66] Perrone C. E. , Shao L., Williams G. M. (1998). Effect of rodent hepatocarcinogenic peroxisome proliferators on fatty acyl-CoA oxidase, DNA synthesis, and apoptosis in cultured human and rat hepatocytes. Toxicol. Appl. Pharmacol. 150, 277–286. 9653058 10.1006/taap.1998.8413

[kfad004-B67] Peters J. M. , Aoyama T., Cattley R. C., Nobumitsu U., Hashimoto T., Gonzalez F. J. (1998). Role of peroxisome proliferator-activated receptor alpha in altered cell cycle regulation in mouse liver. Carcinogenesis 19, 1989–1994. 9855014 10.1093/carcin/19.11.1989

[kfad004-B68] Pham L. L. , Borghoff S. J., Thompson C. M. (2020). Comparison of threshold of toxicological concern (TTC) values to oral reference dose (RFD) values. Regul. Toxicol. Pharmacol. 113, 104651. 32229245 10.1016/j.yrtph.2020.104651

[kfad004-B69] Rakhshandehroo M. , Hooiveld G., Müller M., Kersten S. (2009). Comparative analysis of gene regulation by the transcription factor pparalpha between mouse and human. PLoS One 4, e6796. 19710929 10.1371/journal.pone.0006796 PMC2729378

[kfad004-B70] Ratajczak W. , Atkinson S. D., Kelly C. (2022). The TWEAK/Fn14/CD163 axis-implications for metabolic disease. Rev. Endocr. Metab. Disord. 23, 449–462. 34542797 10.1007/s11154-021-09688-4 PMC9156485

[kfad004-B71] Rodríguez A. , Catalán V., Gómez-Ambrosi J., Frühbeck G. (2006). Role of aquaporin-7 in the pathophysiological control of fat accumulation in mice. FEBS Lett. 580, 4771–4776. 16919625 10.1016/j.febslet.2006.07.080

[kfad004-B72] Rosen M. B. , Das K. P., Rooney J., Abbott B., Lau C., Corton J. C. (2017). PPARα-independent transcriptional targets of perfluoroalkyl acids revealed by transcript profiling. Toxicology 387, 95–107. 28558994 10.1016/j.tox.2017.05.013 PMC6129013

[kfad004-B73] Sonich-Mullin C. , Fielder R., Wiltse J., Baetcke K., Dempsey J., Fenner-Crisp P., Grant D., Hartley M., Knaap A., Kroese D. et al; International Programme on Chemical Safety. (2001). IPCS conceptual framework for evaluating a mode of action for chemical carcinogenesis. Regul. Toxicol. Pharmacol. 34, 146–152. 11603957 10.1006/rtph.2001.1493

[kfad004-B74] Tahri-Joutey M. , Andreoletti P., Surapureddi S., Nasser B., Cherkaoui-Malki M., Latruffe N. (2021). Mechanisms mediating the regulation of peroxisomal fatty acid beta-oxidation by PPARα. Int. J. Mol. Sci. 22, 8969. 34445672 10.3390/ijms22168969 PMC8396561

[kfad004-B75] Thompson C. M. , Fitch S. E., Ring C., Rish W., Cullen J. M., Haws L. C. (2019). Development of an oral reference dose for the perfluorinated compound GenX. J. Appl. Toxicol. 39, 1267–1282. 31215065 10.1002/jat.3812 PMC6771874

[kfad004-B91] Thompson C. M. , Heintz M. M., Wolf J. C., Cheru R., Haws L. C., Cullen J. M. Assessment of mouse liver histopathology following exposure to HFPO-DA with emphasis on understanding mechanisms of hepatocellular death. Toxicol. Pathol. Forthcoming. 10.1177/01926233231159078 PMC10278389 36987989

[kfad004-B77] Thoolen B. , Maronpot R. R., Harada T., Nyska A., Rousseaux C., Nolte T., Malarkey D. E., Kaufmann W., Küttler K., Deschl U., et al (2010). Proliferative and nonproliferative lesions of the rat and mouse hepatobiliary system. Toxicol. Pathol. 38, 5s–81s. 21191096 10.1177/0192623310386499

[kfad004-B78] USEPA. (2005). Guidelines for carcinogen risk assessment, EPA/630/p-03/001f. Risk assessment forum, US Environmental Protection Agency, Washington DC, March 2005.

[kfad004-B79] USEPA. (2021). Human health toxicity values for hexafluoropropylene oxide (HFPO) dimer acid and its ammonium salt (casrn 13252-13-6 and casrn 62037-80-3) also known as “GenX chemicals” EPA document number: 822r-21-010. U.S. Environmental Protection Agency Office of Water (4304t) Health and Ecological Criteria Division. Washington DC, USA.

[kfad004-B80] USFDA. (2018). Tricor (fenofibrate) tablet, for oral use. Reference id: 4410834. U.S. Food and Drug Administration. Available at: https://www.Accessdata.Fda.Gov/drugsatfda_docs/label/2019/021656s029lbl.Pdf. Accessed: September 15, 2022.

[kfad004-B81] Wang G. , Duan J., Pu G., Ye C., Li Y., Xiu W., Xu J., Liu B., Zhu Y., Wang C. (2022a). The Annexin A2-Notch regulatory loop in hepatocytes promotes liver fibrosis in NAFLD by increasing osteopontin expression. Biochim. Biophys. Acta. Mol. Basis Dis. 1868, 166413. 35413401 10.1016/j.bbadis.2022.166413

[kfad004-B82] Wang J. , Wang X., Sheng N., Zhou X., Cui R., Zhang H., Dai J. (2017). RNA-sequencing analysis reveals the hepatotoxic mechanism of perfluoroalkyl alternatives, HFPO2 and HFPO4, following exposure in mice. J. Appl. Toxicol. 37, 436–444. 27553808 10.1002/jat.3376

[kfad004-B83] Wang W. , Xia X. R., Kong XQ, Shea D. (2022b). Concentrations of hexafluoropropylene oxide dimer acid (HFPO-DA) in food and environmental media in the United States. Statera environmental. Available at: https://static1.squarespace.com/static/5759f142c6fc085e2d4b8854/t/62a75184c958fc5231e3b225/1655132548884/HFPO-DA_Data_Reports_US_China_Statera_May_2022.pdf. Accessed: July 19, 2022.

[kfad004-B84] Wang Z. , Cousins I. T., Scheringer M., Hungerbühler K. (2013). Fluorinated alternatives to long-chain perfluoroalkyl carboxylic acids (PFCAS), perfluoroalkane sulfonic acids (PFSAS) and their potential precursors. Environ. Int. 60, 242–248. 24660230 10.1016/j.envint.2013.08.021

[kfad004-B85] Ward J. M. , Hagiwara A., Anderson L. M., Lindsey K., Diwan B. A. (1988). The chronic hepatic or renal toxicity of di(2-ethylhexyl) phthalate, acetaminophen, sodium barbital, and phenobarbital in male B6c3F1 mice: Autoradiographic, immunohistochemical, and biochemical evidence for levels of DNA synthesis not associated with carcinogenesis or tumor promotion. Toxicol. Appl. Pharmacol. 96, 494–506. 3206528 10.1016/0041-008x(88)90009-9

[kfad004-B86] Xiao S. , Anderson S. P., Swanson C., Bahnemann R., Voss K. A., Stauber A. J., Corton J. C. (2006). Activation of peroxisome proliferator-activated receptor alpha enhances apoptosis in the mouse liver. Toxicol. Sci. 92, 368–377. 16687391 10.1093/toxsci/kfl002

[kfad004-B87] Xie C. , Takahashi S., Brocker C. N., He S., Chen L., Xie G., Jang K., Gao X., Krausz K. W., Qu A., et al (2019). Hepatocyte peroxisome proliferator-activated receptor α regulates bile acid synthesis and transport. Biochim. Biophys. Acta. Mol. Cell Biol. Lipids 1864, 1396–1411. 31195146 10.1016/j.bbalip.2019.05.014 PMC7423166

[kfad004-B88] Yang Q. , Nagano T., Shah Y., Cheung C., Ito S., Gonzalez F. J. (2008). The PPAR alpha-humanized mouse: A model to investigate species differences in liver toxicity mediated by PPAR alpha. Toxicol. Sci. 101, 132–139. 17690133 10.1093/toxsci/kfm206 PMC2197159

[kfad004-B89] Zhang Y. , Zhang Y., Klaassen C. D., Cheng X. (2018). Alteration of bile acid and cholesterol biosynthesis and transport by perfluorononanoic acid (PFNA) in mice. Toxicol. Sci. 162, 225–233. 29112762 10.1093/toxsci/kfx237 PMC6693384

[kfad004-B90] Zisser A. , Ipsen D. H., Tveden-Nyborg P. (2021). Hepatic stellate cell activation and inactivation in NASH-fibrosis-roles as putative treatment targets? Biomedicines 9, 365. 33807461 10.3390/biomedicines9040365 PMC8066583

