# Supplementary material for: Assessment of the mode of action underlying development of liver lesions in mice following oral exposure to HFPO-DA and relevance to humans
Source: Toxicol Sci. 2023 Jan 11;192(1):15–29. doi: 10.1093/toxsci/kfad004 (PMC10025879; doi:10.1093/toxsci/kfad004)
Supplement: kfad004_Supplementary_Data [file kfad004_supplementary_data.docx]

**Supplementary Table and Figure**

**Table S1.** Comparison of enriched gene sets for general peroxisome/PPAR signaling and PPARα- versus PPARγ-specific gene sets in livers of HFPO-DA-exposed mice.

| **Groups of Canonical Pathways** | **Gene Set** | **Sex** | **HFPO-DA mg/kg-bw/day** | **90-day Subchronic Toxicity Study**  **(Chappell et al., 2020)^a^** | | **Reproductive/developmental Toxicity Study**  **(Heintz et al., 2022)** | |
| --- | --- | --- | --- | --- | --- | --- | --- |
|  |  |  |  | **Adjusted P-value^b,c^** | **Overall Direction** | **Adjusted**  **P-value^b,c^** | **Overall Direction** |
| **General PPAR / Peroxisomal Signaling** | **KEGG Peroxisome** | Female | 0.1 | 1 | NS | 1 | NS |
|  |  |  | 0.5 | **4.82E-10** | Up | **6.8911E-09** | Up |
|  |  |  | 5 | **5.91E-20** | Up | **4.6811E-14** | Up |
|  |  | Male | 0.1 | 1 | NS | 1 | NS |
|  |  |  | 0.5 | **3.15E-11** | Up | **1.8276E-09** | Up |
|  |  |  | 5 | **4.70E-10** | Up | **4.9931E-12** | Up |
|  | **KEGG PPAR Signaling** | Female | 0.1 | 1 | NS | 1 | NS |
|  |  |  | 0.5 | **1.38E-10** | Up | **3.1184E-09** | Up |
|  |  |  | 5 | **1.38E-17** | Up | **1.6363E-17** | Up |
|  |  | Male | 0.1 | 1 | NS | 1 | NS |
|  |  |  | 0.5 | **1.04E-07** | Up | **4.4076E-14** | Up |
|  |  |  | 5 | **1.76E-16** | Up | **1.8259E-12** | Up |
|  | **REACTOME Peroxisomal Lipid Metabolism** | Female | 0.1 | 1 | NS | 1 | NS |
|  |  |  | 0.5 | **7.08E-09** | Up | **2.388E-08** | Up |
|  |  |  | 5 | **1.31E-08** | Up | **3.9408E-09** | Up |
|  |  | Male | 0.1 | 1 | NS | 1 | NS |
|  |  |  | 0.5 | **4.56E-07** | Up | **4.0131E-08** | Up |
|  |  |  | 5 | **6.80E-07** | Up | **3.9031E-06** | Up |
|  | **REACTOME β-Oxidation of Very Long-Chain Fatty Acids** | Female | 0.1 | 1 | NS | 1 | NS |
|  |  |  | 0.5 | **1.059E-07** | Up | **1.52E-08** | Up |
|  |  |  | 5 | **1.635E-06** | Up | **2.97E-05** | Up |
|  |  | Male | 0.1 | 1 | NS | 1 | NS |
|  |  |  | 0.5 | **3.47E-06** | Up | **4.68E-06** | Up |
|  |  |  | 5 | **3.51E-06** | Up | **6.19E-05** | Up |
|  | **WP PPAR Signaling** | Female | 0.1 | 1 | NS | 1 | NS |
|  |  |  | 0.5 | **2.73E-09** | Up | **5.542E-08** | Up |
|  |  |  | 5 | **2.14E-16** | Up | **1.0497E-16** | Up |
|  |  | Male | 0.1 | 1 | NS | 1 | NS |
|  |  |  | 0.5 | **8.61E-07** | Up | **7.0315E-13** | Up |
|  |  |  | 5 | **1.56E-14** | Up | **5.5036E-12** | Up |
| **PPARα signaling** | **BIOCARTA PPARα Pathway** | Female | 0.1 | 1 | NS | 1 | NS |
|  |  |  | 0.5 | **0.00471145** | Up | **0.00078767** | Up |
|  |  |  | 5 | **0.00800287** | Up | **0.00847825** | Up |
|  |  | Male | 0.1 | 1 | NS | 1 | NS |
|  |  |  | 0.5 | **0.01387067** | Up | **0.00769699** | Up |
|  |  |  | 5 | **0.01350603** | Up | 0.4160496 | NS |
|  | **REACTOME Regulation of Lipid Metabolism by PPARα** | Female | 0.1 | 1 | NS | 1 | NS |
|  |  |  | 0.5 | 0.08997489 | NS | **0.01161897** | Up |
|  |  |  | 5 | **0.00067361** | Up | **0.00573576** | Up |
|  |  | Male | 0.1 | 1 | NS | 1 | NS |
|  |  |  | 0.5 | **0.01557152** | Up | **2.3197E-05** | Up |
|  |  |  | 5 | **0.00068848** | Up | **0.00826333** | Up |
|  | **WP PPARα Pathway** | Female | 0.1 | 1 | NS | 1 | NS |
|  |  |  | 0.5 | **1.75E-07** | Up | **7.902E-07** | Up |
|  |  |  | 5 | **9.51E-08** | Up | **1.8792E-08** | Up |
|  |  | Male | 0.1 | 1 | NS | 1 | NS |
|  |  |  | 0.5 | **0.00076206** | Up | **1.6442E-08** | Up |
|  |  |  | 5 | **0.00175762** | Up | **9.3209E-05** | Up |
| **PPARγ signaling** | **BIOCARTA PPARγ Pathway** | Female | 0.1 | 1 | NS | 1 | NS |
|  |  |  | 0.5 | 1 | NS | 1 | NS |
|  |  |  | 5 | 0.719265585 | NS | 1 | NS |
|  |  | Male | 0.1 | 1 | NS | 1 | NS |
|  |  |  | 0.5 | 1 | NS | 1 | NS |
|  |  |  | 5 | 0.9468714 | NS | 0.4668795 | NS |
|  | **WP HIF1α and PPARγ Regulation of Glycolysis** | Female | 0.1 | 1 | NS | 1 | NS |
|  |  |  | 0.5 | 0.86272933 | NS | 1 | NS |
|  |  |  | 5 | 0.09766636 | NS | 1 | NS |
|  |  | Male | 0.1 | 1 | NS | 1 | NS |
|  |  |  | 0.5 | 0.1285843 | NS | 1 | NS |
|  |  |  | 5 | 0.7833859 | NS | 0.7100514 | NS |

^a^ Updated gene set enrichment analysis of transcriptomic data from Chappell et al. (2020) using methods

described in Heintz et al. (2022).

^b^ **Bold** formatting indicates significant adjusted P-value (i.e., FDR ≤ 0.05); NS = Not significant (FDR > 0.05)

^c^ Enrichment of gene sets was determined using the hypergeometric test as described in Heintz et al. (2022).

**Figure S1.** Significant differentially expressed genes that are a part of the KEGG PPAR signaling network in livers of HFPO-DA exposed male and female mice from Heintz et al. (2022) and Chappell et al. (2020). Ligands, transcription factors, and genes, as related to PPAR α/γ/δ signaling, are shown according to the KEGG database. Individual PPAR signaling genes that are significantly differentially expressed in one or both studies are notated by color-coded shapes by study, sex, and concentration. Arrows show the target genes corresponding to each PPAR isoform: green = PPARα, purple = PPARδ, red = PPARγ. Figure adapted from Chappell et al. (2020) and Heintz et al. (2022). KEGG, Kyoto Encyclopedia of Genes and Genomes; PPAR, peroxisome proliferator-activated receptor.
